# Identifying novel genes and chemicals related to nasopharyngeal cancer in a heterogeneous network

**DOI:** 10.1038/srep25515

**Published:** 2016-05-05

**Authors:** Zhandong Li, Lifeng An, Hao Li, ShaoPeng Wang, You Zhou, Fei Yuan, Lin Li

**Affiliations:** 1 College of Biology and Food Engineering, Jilin Engineering Normal University, Changchun, China; 2 Department of Otorhinolaryngology and Head & Neck, China-Japan Union Hospital attached to Jilin University, Changchun, China; 3 School of Life Sciences, Shanghai University, Shanghai, China; 4 Institute of Health Sciences, Shanghai Institutes for Biological Sciences, Chinese Academy of Sciences & Shanghai Jiao Tong University School of Medicine, Shanghai, China

## Abstract

Nasopharyngeal cancer or nasopharyngeal carcinoma (NPC) is the most common cancer originating in the nasopharynx. The factors that induce nasopharyngeal cancer are still not clear. Additional information about the chemicals or genes related to nasopharyngeal cancer will promote a better understanding of the pathogenesis of this cancer and the factors that induce it. Thus, a computational method NPC-RGCP was proposed in this study to identify the possible relevant chemicals and genes based on the presently known chemicals and genes related to nasopharyngeal cancer. To extensively utilize the functional associations between proteins and chemicals, a heterogeneous network was constructed based on interactions of proteins and chemicals. The NPC-RGCP included two stages: the searching stage and the screening stage. The former stage is for finding new possible genes and chemicals in the heterogeneous network, while the latter stage is for screening and removing false discoveries and selecting the core genes and chemicals. As a result, five putative genes, CXCR3, IRF1, CDK1, GSTP1, and CDH2, and seven putative chemicals, iron, propionic acid, dimethyl sulfoxide, isopropanol, erythrose 4-phosphate, β-D-Fructose 6-phosphate, and flavin adenine dinucleotide, were identified by NPC-RGCP. Extensive analyses provided confirmation that the putative genes and chemicals have significant associations with nasopharyngeal cancer.

The nasopharynx is the upper part of the pharynx, which is a tube that starts behind the nose and ends at the top of the trachea and esophagus^1^. Nasopharyngeal cancer, or nasopharyngeal carcinoma (NPC), is classified as a malignant tumor. Cervical lymphadenopathy occurs in many patients. Other symptoms include trouble breathing or speaking, hearing loss and nose bleeding. After metastasis, bone pain and organ dysfunction may occur. NPC has three types: squamous cell carcinoma (Type I); keratinizing undifferentiated carcinoma (Type II); and nonkeratinizing undifferentiated carcinoma (Type III)^2^. NPC is uncommon in western countries, but it is quite common in the southern part of China, especially in Guangdong Province^3^. Therefore, it is also called Cantonese cancer.

What exactly causes nasopharyngeal cancer is still unclear. Three main factors being studied are viruses, environmental influences and heredity^4^. Epstein-Barr virus (EBV) has been strongly linked to NPC. Type III is the most common NPC type, and it is most strongly associated with EBV^5^. Inhibition of the LKB1-AMPK pathway by the EBV-encoded LMP1 was found to promote proliferation and transformation of nasopharyngeal epithelial cells^6^. EBV is one of the most common viruses found in the human body, but not everyone who has EBV will get NPC. Human papillomavirus (HPV) may also be associated with NPC^7^. Some small chemicals have been studied. Salt-cured fish and meat are common dietary components in Asia. The volatile nitrosamines contained in the food can increase the chance of NPC^3^,^8^.

The functions of p53 and the retinoblastoma-related gene Rb2/p130 have been studied in nasopharyngeal cancer cell lines^9^,^10^. DNA and mRNA expression profiling analyses were used to evaluate the amplification and deletion of NPC-related genes^11^. Epigenetic alterations such as DNA methylation have the potential to become detection markers and prognostic markers. A survey reported differential methylation of several candidate tumor suppressor genes in NPC compared with controls^12^. Some microRNAs were found to play critical roles in NPC progression. miR-218 suppressed NPC progression through down-regulation of Survivin and the SLIT2-ROBO1 pathway^13^. miR-26a inhibited cell growth and tumorigenesis of NPC through repression of EZH2^14^.

Physical examination of the nose and throat, biopsy, MRI, CT/PET scan and an EBV blood test may be used to diagnose nasopharyngeal cancer^1^. Treatments include surgery, chemotherapy and radiotherapy. Immune-based therapies may be combined for EBV-associated NPC^15^.

As discussed above, the pathogenesis of nasopharyngeal cancer is very complicated. Most studies have focused on viruses that may cause NPC, and the identification of affected genes is far from complete. Small biological molecules require more attention. In recent years, new computational methods and tools have been developed to study protein-related or gene-related problems in protein-protein interaction networks^16^,^17^,^18^ and chemical-related problems in chemical-chemical interaction networks^19^,^20^. Utilizing another network of protein-chemical interactions, a heterogeneous network can be constructed, which provides a novel background to identify disease genes and chemicals simultaneously. This heterogeneous network has already been used to search for new candidate genes and chemicals contributing to esophageal cancer^21^ and prostate cancer^22^, and it has provided some interesting discoveries. In this study, we utilized this heterogeneous network and developed a more strict method, namely the NPC-RGCP (NPC-related Gene and Chemical Prediction) method, to identify novel NPC-related genes and chemicals. The NPC-RGCP included two stages: the searching stage and the screening stage. In the searching stage, a number of candidate genes and chemicals were searched in the heterogeneous network based on the known NPC-related genes and chemicals. In the screening stage, they were filtered by a list of rules. The remaining candidate genes and chemicals are believed to have strong associations with NPC, and this was confirmed by extensive analyses.

## Materials and Methods

### Genes related to NPC

The NPC-related genes were accessed in the following three ways: (1) A total of 27 reviewed NPC-related genes were collected from UniProt (http://www.uniprot.org/, UniProt Release 2014_4), where “human”, “nasopharyngeal cancer” and “reviewed” were used as the keywords to search the UniProt database; (2) Eighteen NPC-related genes were obtained from the TSGene Database (Tumor Suppressor Gene Database, http://bioinfo.mc.vanderbilt.edu/TSGene/cancer_type.cgi)^23^, where the Entrez IDs were converted to the official symbols; and (3) A total of 143 NPC-related genes were retrieved from the NCI (National Cancer Institute, https://gforge.nci.nih.gov, released 2009.6) database. We combined all of these genes and obtained 179 NPC-related genes. These genes were mapped onto their Ensembl IDs, and those that did not occur in the constructed heterogeneous network were removed. A total of 132 Ensembl IDs were finally accessed, and these comprised the set *S*_*p*_ and are shown in Supplementary Material I.

### Chemicals related to NPC

The NPC-related chemicals were extracted from the CTD database^24^ (Comparative Toxicogenomics Database, accessed on July 2, 2014) by searching Nasopharyngeal Neoplasms and Nasopharyngeal carcinoma. The search results were displayed on the following two websites: (1) http://ctdbase.org/detail.go?type=disease&acc=MESH:D009303&view=chem; (2) http://ctdbase.org/detail.go?type=disease&acc=MESH:C538339&view=chem. Only the chemicals with direct evidence of associations with nasopharyngeal cancer, such as “marker”, “mechanism” or “therapeutic”, were selected, resulting in sixteen chemicals. These sixteen chemicals were mapped onto their PubChem IDs, and those without PubChem IDs were removed, resulting in thirteen chemicals remaining. Finally, we removed the PubChem IDs that did not occur in the constructed heterogeneous network, resulting in eight PubChem IDs. These eight PubChem IDs comprised the set *S*_*c*_ and are shown in Table 1.

### Heterogeneous network

The heterogeneous network integrated three types of interaction information: chemical-chemical interactions, protein-chemical interactions, and protein-protein interactions.

The chemical-chemical interaction information was retrieved from STITCH (version 4.0, http://stitch.embl.de/)^25^. The obtained file “chemical_chemical.links.v4.0.tsv.gz” contains a large number of chemical-chemical interactions. For each interaction, there are two chemicals, represented by PubChem IDs, and one score with a range between 1 and 999 to indicate the strength of the interaction. For later formulation, let us denote the score of the interaction between chemicals *c*_1_ and *c*_2_ by *Q*_*cc*_(*c*_1_, *c*_2_).

The protein-chemical interaction information was also accessed from STITCH. In the obtained file “protein_chemical.links.v4.0.tsv.gz”, there are many protein-chemical interactions. Each interaction consists of one protein, represented by an Ensembl ID, one chemical, represented by a PubChem ID, and one score with a range between 1 and 999 to indicate the strength of the interaction. Here, we extracted the human protein-chemical interactions. For protein *p* and chemical *c*, *Q*_*pc*_(*p*, *c*) represented the score of the protein-chemical interaction of *p* and *c*.

The protein-protein interaction information was downloaded from STRING (version 9.1, http://www.string-db.org/). From the obtained file “protein.links.v9.1.txt.gz”, we extracted the human protein-protein interactions by taking lines beginning with “9606”. In each interaction, there are two proteins, represented by Ensembl IDs, and one score with a range between 1 and 999 to represent the strength of the interaction. The score of the interaction between proteins *p*_1_ and *p*_2_ was denoted by *Q*_*pp*_(*p*_1_, *p*_2_) in this study.

**Figure 1:**
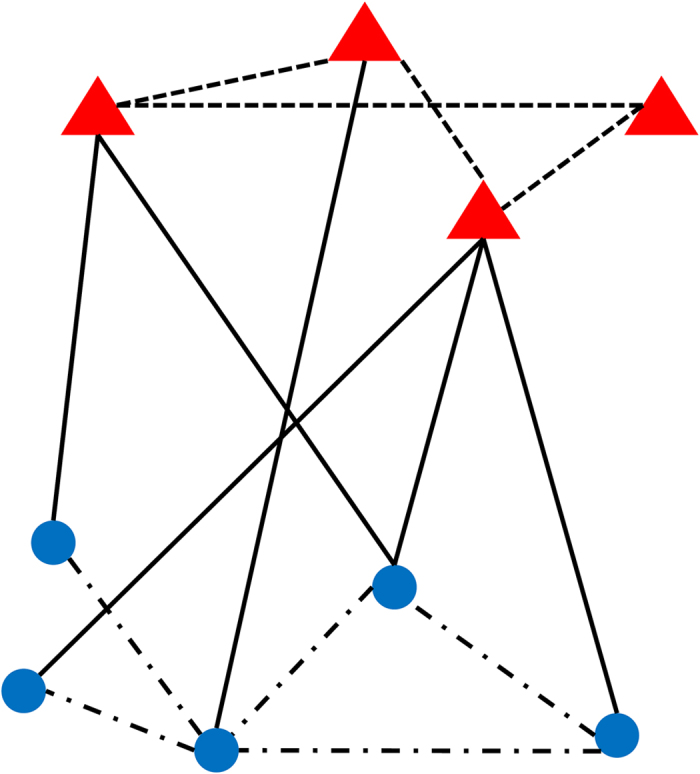
An example showing the structure of the heterogeneous network. The triangles represent proteins, the dots represent chemicals. The sold lines represent protein-chemical interactions, the dashed lines represent protein-protein interactions, and the dashed dotted lines represent chemical-chemical interactions.

Because the number of chemicals reported in STITCH are too large and our computational power is limited, we only took the chemicals that have records in another database, the Kyoto Encyclopedia of Genes and Genomics (KEGG), *i.e.*, the chemical-chemical interactions in which at least one chemical has no records in KEGG and the protein-chemical interactions in which the chemical has no records in KEGG were discarded in this study. In the heterogeneous network, chemicals and proteins occurring in at least one type of the obtained interaction information were taken as nodes. Two nodes were adjacent if and only if the corresponding chemicals/proteins can comprise an interaction. An example is shown in Fig. 1. The interaction score was added into the heterogeneous network as the edge weight. Because the maximum interaction score is 999 and the shortest path algorithm requires an edge with a low weight indicates strong correlations between its endpoints, each edge *e* connecting nodes *n*_1_ and *n*_2_ was assigned a weight in the following manner:


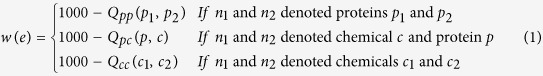


The final heterogeneous network contained 35,842 nodes, where 20,770 nodes represented proteins and 15,072 nodes represented chemicals. The number of edges in the heterogeneous network was 3,046,625, where 398,701 edges represented chemical-chemical interactions, 222,610 edges represented protein-chemical interactions and 2,425,314 edges represented protein-protein interactions.

### NPC-RGCP method

In the heterogeneous network described in Section “Heterogeneous network”, a computational method, namely the NPC-RGCP method, was built to identify novel NPC-related genes and chemicals. The method consisted of two stages: (1) the searching stage: finding new possible NPC-related genes and chemicals in the heterogeneous network; and (2) the screening stage: excluding false positives obtained in the searching stage and selecting core genes and chemicals.

#### Searching stage

It is known that chemicals or proteins that interact are highly likely to share the same functions^17^,^18^,^19^,^26^,^27^,^28^,^29^,^30^. Compared with chemicals or proteins in interactions with low scores (corresponding edges have high weights in the heterogeneous network), chemicals or proteins in interactions with high scores are more likely to have tighter associations with each other. Therefore, it can be inferred that chemicals or proteins of the inner nodes in a shortest path may have the same functions as the chemicals or proteins of the end-points of the path. Thus, all shortest paths connecting any two NPC-related genes or chemicals were searched in the heterogeneous network. The inner nodes of these paths were extracted, and the corresponding genes or chemicals that were not NPC-related genes or chemicals were considered candidates for NPC. For convenience, they were called the shortest path genes or chemicals. Furthermore, a measurement, called betweenness, was assigned to each shortest path gene or chemical, which was defined as the number of shortest paths containing it. Betweenness can measure the strength between the shortest path genes or chemicals and NPC. Generally, a shortest path gene or chemical with a high betweenness may have strong associations with NPC.

#### Screening stage

The searching stage provided a number of shortest path genes and chemicals. However, some of them may be false positives. For example, some shortest path genes and chemicals have universal associations with other chemicals and genes. Their corresponding nodes in the constructed heterogeneous network are general hubs, indicating that they may lie in a shortest path connecting any two nodes with high probabilities. If we randomly selected some genes and chemicals in the network to replace the NPC-related genes and chemicals, they may still be selected as the shortest path genes or chemicals. To account for this, a permutation test was performed. A total of 1,000 sets were randomly produced, where each set contained the same number of genes in *S*_*p*_ and the same number of chemicals in *S*_*c*_. For each randomly produced set, all shortest paths connecting any two members in the set were searched in the heterogeneous network. The betweenness of each shortest path gene or chemical was counted based on these paths. Accordingly, another measurement, called permutation FDR, was calculated as follows:


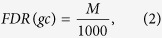


where *gc* represents a shortest path gene or chemical and M represents the number of randomly produced sets that produce larger betweenness than that produced by 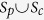. It is easy to recognize that a shortest path gene or chemical with a high permutation FDR means it is a general hub in the network and has few associations with NPC. Because 0.05 is always used as the critical point of significance level of the test, it was set for the permutation FDR to exclude shortest path genes or chemicals with high permutation FDRs.

The permutation test mentioned above can only exclude universal genes and chemicals among the shortest path genes and chemicals. To extract the most possible genes and chemicals among the remaining shortest path genes or chemicals, a further screening method is necessary.

It has been reported in many previous studies that proteins that can interact with each other are more likely to share similar functions than those that cannot^26^,^27^,^28^,^29^,^30^. In addition, the strength of an interaction is indicated by its score. Proteins in an interaction with a high score have a higher likelihood of sharing similar functions than those in an interaction with a low score. Thus, the protein interactions and their scores can help us further select the most possible genes. For each shortest path gene, we calculated its maximum interaction score (MIS) as follows:


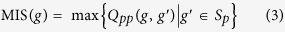


On the other hand, protein structure is a type of classic information used to identify the functions of proteins. The widely accepted fact is that proteins with similar structures are more likely to share similar functions. Thus, another screening rule was built based on the sequence similarity between the shortest path genes and NPC-related genes. The basic local alignment search tool (BLAST)^31^ was used to search the local similarities between the protein sequences. For the formulation, let us denote the alignment score between proteins *p*_1_ and *p*_2_ by *Q*_*A*_(*p*_1_, *p*_2_). For each shortest path gene, we calculated its maximum alignment score (MAS) as follows:


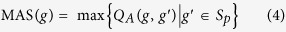


The shortest path genes with a MIS larger than or equal to 900 and a MAS no less than 90 were selected as the putative NPC-related genes. We selected 900 as the threshold of MIS and 90 as the threshold of MAS because 900 is defined as the cutoff of the highest confidence level in STRING and 90 is deemed to be an important score for implying homology of two proteins.

For the shortest path chemicals, a similar screening method was constructed. It has been reported in some studies that chemicals that interact always share common functions^19^,^20^,^26^. Considering that the interaction score can induce the result that chemicals in an interaction with a high score are more likely to share common functions than those in an interaction with a low score, we calculated the maximum interaction score (MIS) for each shortest path chemical as follows:


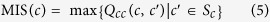


The shortest path chemicals with a MIS larger than or equal to 900 were selected as the putative NPC-related chemicals. The reason why we selected 900 as the threshold of MIS is that 900 is defined as the cutoff of the highest confidence level in STITCH.

In summary, the pseudocode of the algorithm for NPC-RGCP is in Table 2.

## Results

**Figure 2:**
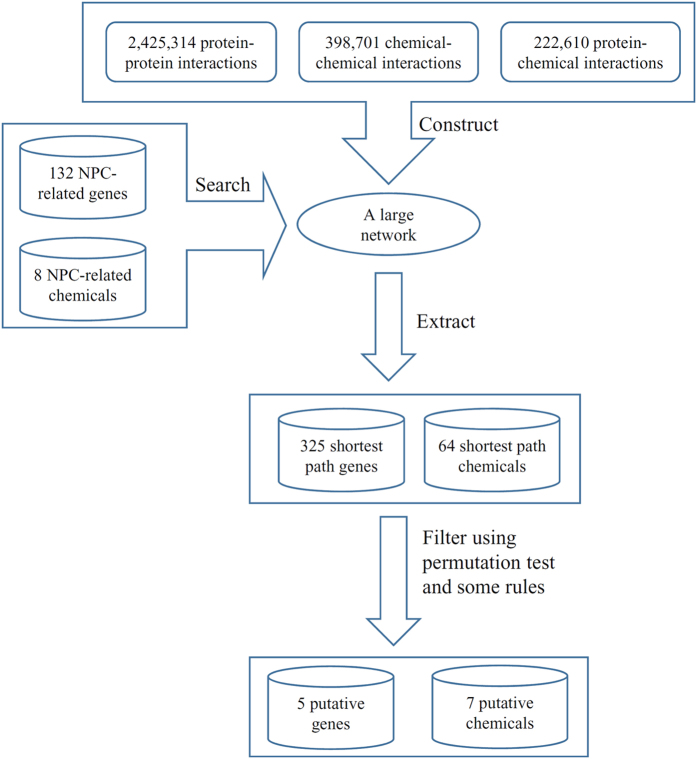
A figure illustrating the procedures of the NPC-RGCP method and its results.

The NPC-RGCP method described above was used to identify novel NPC-related genes and chemicals. The whole procedures and results are summarized in Fig. 2. This section would give a detailed description of the results.

### Results of the searching stage of the NPC-RGCP method

According to the searching stage of the algorithm for NPC-RGCP, all shortest paths connecting any two NPC-related genes or chemicals were searched in the heterogeneous network. Based on these paths, we extracted 331 Ensembl IDs and 64 PubChem IDs. The 331 Ensembl IDs were mapped onto their gene symbols, resulting in 325 gene symbols. Thus, in this stage, we obtained 325 shortest path genes and 64 shortest path chemicals, which are shown in Supplementary Material II. The betweenness of these shortest path genes and chemicals were also calculated and are shown in Supplementary Material II.

### Results of the screening stage of the NPC-RGCP method

The screening stage of the NPC-RGCP method was used to filter false positives and select key genes and chemicals. First, the permutation test was executed to control for universal genes and chemicals in the heterogeneous network among the shortest path genes and chemicals obtained in Section “Results of the searching stage of the NPC-RGCP method”. For each shortest path gene and chemical, we calculated its permutation FDR, which is shown in Supplementary Material III. Second, for shortest path genes, the MISs and MASs (cf. Eqs 3 and 4) were calculated and are shown in Supplementary Material III. For shortest path chemicals, we calculated the MISs using Eq. 5, and these are also shown in Supplementary Material III. Finally, we selected the shortest path genes with permutation FDRs smaller than 0.05, MISs no less than 900 and MASs no less than 90 and the shortest path chemicals with permutation FDRs smaller than 0.05 and MISs no less than 900, resulting in five genes and seven chemicals. These genes and chemicals are considered to be significant for NPC and termed the putative genes and chemicals for NPC. The detailed information of the five putative genes and seven putative chemicals is listed in Tables 3 and 4, respectively.

## Discussion

The NPC-RGCP provided five putative genes and seven putative chemicals for NPC. In this section, these genes and chemicals are extensively discussed.

### Analysis of putative genes

Five putative genes, listed in Table 3, were identified by NPC-RGCP. We analyzed them one by one to confirm their likelihood of being NPC-related genes.

### CXCR3

The betweenness and permutation FDR of CXCR3 (chemokine receptor 3, Ensembl ID: ENSP00000362795) were 136 and 0.015, respectively (row 1 of Table 3). CXCR3, also known as GPR9 and CD183, is a seven-transmembrane G protein-coupled receptor in the CXC chemokine receptor family. It is expressed largely on activated dendritic cells (DCs), T-lymphocytes, natural killer (NK) cells, and several epithelial cells. Numerous studies have demonstrated key roles for CXCR3 in T cell trafficking and function by promoting the interaction of T cells with antigen-presenting cells (APCs) as well as generating effector and memory cells^32^. More recent studies have also suggested the ambiguous role of CXCR3 in carcinogenesis. On the one hand, CXCR3 ligands can effectively inhibit lymphangiogenesis and serve as anti-tumor agents^33^, while, on the other hand, high CXCR3 expression is associated with lymph node invasion and poor prognosis. Given the involvement of CXCR3 in the antigen processing and presentation pathway, which takes over the crucial position in NPC progression as mentioned previously, and its potential endogenous tumor control, it has been speculated that CXCR3 may be an essential factor contributing to the function of the nasopharynx whose dysregulation may result in carcinogenesis.

### IRF1

The betweenness and permutation FDR of IRF1 (interferon regulatory factor 1, Ensembl ID: ENSP00000245414) were 151 and 0.013, respectively (row 2 of Table 3). IRF1, the first member of the interferon regulatory transcription factor family to be identified, has been extensively characterized at the molecular level. Initially isolated because of its affinity to particular DNA sequences in the cytokine Interferon (IFN)-β, IRF1 was subsequently shown to act as a transcriptional activator or repressor of multiple target genes, including trans-activating p53 by recruiting its co-factor p300^34^. Multiple lines of evidence have also emphasized the influence of IRF1 on the immune response, DNA damage, apoptosis regulation and tumor suppression^35^. Recent studies have demonstrated that IRF1 and MHC class I molecules were modulated by miR-9 in human NPC cells^36^, linking inflammation with cancer, which might facilitate the pathogenesis of NPC. This mechanism remains to be fully clarified.

### CDK1

The betweenness and permutation FDR of CDK1 (cyclin-dependent kinase 1, Ensembl ID: ENSP00000306043) were 667 and 0.021, respectively (row 3 of Table 3). CDK1 is a highly conserved serine/threonine kinase, and it forms complexes with its cyclin partners that phosphorylate a wide variety of target substrates, playing key roles in cell cycle progression and regulation. Previous studies have observed universal overactivity of the cyclin-dependent kinases caused by diverse genetic and epigenetic events in human cancers, inhibition of which can lead to both cell cycle arrest and apoptosis^37^. For example, in prostate cancer cells, aberrant activation of CDK1 was found to promote cell proliferation as well as survival by virtue of phosphorylation and inhibition of FOXO1 that prolonged the transition from G2 to M phase and functioned as a tumor suppressor, thus contributing to tumorigenesis. In addition, CDK1 was optionally lethal to MYC-dependent human breast cancer cells, suggesting further investigation of CDK1 inhibition as a potential therapy for this type of breast cancer^38^. Therefore, it has been inferred that CDK1 promotes NPC cell growth by regulating the cell cycle and could be designed as a drug therapeutic target.

### GSTP1

The betweenness and permutation FDR of GSTP1 (glutathione S-transferase pi 1, Ensembl ID: ENSP00000381607) were 131 and 0.019, respectively (row 4 of Table 3). GSTP1 is the most widely distributed member of the glutathione S-transferase (GST) family that catalyzes intracellular detoxification of various electrophiles and plays critical roles in susceptibility to many diseases including cancer. In addition to significant links with prostate cancer, breast cancer, ovarian cancer, and bladder cancer, GSTP1 has been found to be involved in the progression of NPC. The aberrant DNA methylation of GSTP1 contributed to Taxol drug resistance in NPC cells. GSTP1 was also relevant to NPC radioresistance. Moreover, aberrant regulation of the epidermal growth factor receptor (EGFR) signaling pathway was commonly found in NPC cells, which was related to tumor recurrence, metastasis, and poor survival^39^. As an EGFR-regulated protein, GSTP1 has become a new target for NPC therapy. Taken together, an increasing number of studies have proposed the pivotal roles of GSTP1 in NPC, confirming the robustness of our analysis, and further mechanisms need to be explored and experimentally validated.

### CDH2

The betweenness and permutation FDR of CDH2 (cadherin 2, Ensembl ID: ENSP00000269141) were 421 and 0.03, respectively (row 5 of Table 3). CDH2 is a calcium dependent cell-cell adhesion glycoprotein that is required for the establishment of left-right asymmetry. It has been shown to interact with β-catenin in the Wnt signaling pathway, whose dysregulation was associated with a variety of cancers including colorectal carcinoma, hepatocellular carcinoma, lung cancer, and esophageal carcinoma^40^. N-cadherin was up-regulated among multiple cancer cells, providing a mechanism for invasion and metastasis^41^. Additionally, inactivation of N-cadherin was shown to prevent cell migration. Furthermore, recent data has suggested that there is a correlation between the expression of nuclear N-cadherin and poor prognosis of NPC, indicating that N-cadherin is a potential prognostic marker in NPC patients. Consistent with the previously mentioned standpoint, our results again emphasized the crucial roles N-cadherin played in the development and progression of NPC.

### Analysis of putative chemicals

We identified seven putative chemicals by NPC-RGCP (see Table 4). The extensive analysis of these chemicals as novel NPC-related chemicals is described below.

### Iron

The betweenness and permutation FDR of iron (PubChem ID: CID000023925) were 658 and 0.002, respectively (row 1 of Table 4). In vertebrates, hemoglobin and myoglobin are common oxygen transport proteins, in which cellular iron usually forms complexes with molecular oxygen and plays important roles in blood cell maturation and function. A growing number of studies have indicated that abnormal iron homeostasis may result in human diseases. Because iron is readily available from various sources of dietary iron including red meat, fish, fortified bread and beans, several studies have suggested that hemoglobin from red meat may increase the risk of colorectal cancer^42^. Very recent studies have found iron deposition on metaplastic bones in patients with diffuse pulmonary ossification (DPO), which may disrupt the normal mineralization processes of the metaplastic bones. Mutations in TFR2, which mediates cellular uptake of transferrin-bound iron, have been associated with hereditary hemochromatosis type III^43^. Additionally, iron is pervasively found at the active site of many important redox enzymes, whose altered expression has been detected in epithelial ovarian cancer. Our results expanded the connections of iron with cancer and identified iron as a putative carcinogen for NPC, requiring more clinical samples for validation.

### Propionic acid

The betweenness and permutation FDR of propionic acid (PubChem ID: CID000001032) were 139 and <0.001, respectively (row 2 of Table 4). Propionic acid is mainly used as a chemical intermediate in herbicides, cellulose fiber, and pharmaceutical synthesis. A well-documented example of a propionic acid causative disease is a rare inherited genetic disorder called propionic academia. In liver cells, propionate acts as a metabolic toxin through accumulation in the mitochondria as propionyl-CoA as well as tricarboxylic acid (TCA) cycle inhibitors^44^. Continuous propionic academia leads to reversible symptoms such as neurodegeneration, dystonia, cardiomyopathy, and social impairment^44^,^45^. It has also been shown that propionic acid not only induced apoptosis of colorectal carcinoma cells, but also enhanced the expression of the NKG2D ligand in activated T-lymphocytes and cancer cells, representing remarkable immunoregulatory function and cancer prevention ability. In addition to the tumor types discussed above, our results indicated, for the first time, that propionic acid participated in the metabolism of NPC cells. Future research will be required to determine the detailed mechanism.

### Dimethyl sulfoxide

The betweenness and permutation FDR of dimethyl sulfoxide (PubChem ID: CID000000679) were 14 and 0.048, respectively (row 3 of Table 4). Dimethyl sulfoxide (DMSO) is widely used as a polar aprotic solvent with pharmacological actions including bacteriostatic, analgesic, anti-inflammatory, diuresis, and muscle relaxation capabilities. Because of its unique capability to safely penetrate living tissues, DMSO is also used as a carrier to promote the bladder absorption of chemotherapeutic drugs, and it is approved for the treatment of interstitial cystitis. Furthermore, DMSO has been reported to induce growth arrest and cellular differentiation in murine erythroleukemia cells (MELC)^46^, which suggested to us that it might preform similarly in the occurrence of NPC. Therefore, we speculated that DMSO might be a novel key element in NPC through its ability to influence the movement of drugs and alter cell fate.

### Isopropanol

The betweenness and permutation FDR of isopropanol (PubChem ID: CID000003776) were 108 and 0.013, respectively (row 4 Table 4). Isopropanol is manufactured by the direct or indirect hydration of propylene, and it is mainly used for the production of chemicals, pharmaceuticals and cosmetics. Human acute exposure to isopropanol causes symptoms that range from mild irritation to coma or even death. Additionally, an increased incidence of paranasal sinus cancer has been observed in patients who had lifelong exposure to isopropanol, although evidence is inadequate to infer that this chemical is definitely a carcinogenic agent^47^. Aside from the effects of isopropanol on humans, animals with prolonged exposure to isopropanol also suffer from central nervous system depression, liver damage, and unconsciousness. Thus, our results provide additional powerful evidence implying the carcinogenicity of isopropanol in NPC, and more investigations are needed to prove this relationship.

### Erythrose 4-phosphate

The betweenness and permutation FDR of erythrose 4-phosphate (PubChem ID: CID000122357) were 2 and 0.024, respectively (row 5 of Table 4). Erythrose 4-phosphate is part of the pentose phosphate pathway (PPP), which plays pivotal roles in cancer cell metabolism and survival via assisting glycoclastic cancer cells in meeting their anabolic requirements and combating oxidative stress. Prior studies have also suggested the influences of tumor suppressor proteins and oncoproteins, such as p53 and RAS, on PPP, of which the mechanisms included regulating the expression and activities of enzymes that governed PPP modes^48^. Erythrose 4-phosphate is also the inhibitor of phosphoglucose isomerase/autocrine motility factor (PGI/AMF), which is essential to anabolic gluconeogenesis and catabolic glycolysis, regulating cancer cell proliferation and metastasis. These observations corresponded to our GO term analysis, underlining the importance of regulation of cellular metabolism in tumor ontogeny. In summary, erythrose 4-phosphate may also be involved in the metabolic pathway of NPC cells, providing a potentially effective and specific approach for NPC treatment.

### β-D-Fructose 6-phosphate

The betweenness and permutation FDR of β-D-fructose 6-phosphate (PubChem ID: CID000440641) were 139 and 0.001, respectively (row 6 of Table 4). β-D-Fructose 6-phosphate is the β-D-form of fructose 6-phosphate that is commonly found in cells and fructose sugar phosphorylated on carbon 6. It lies within the glycolysis metabolic pathway, in which PGI is responsible for catalyzing the conversion of fructose 6-phosphate and glucose 6-phosphate. Actually, in 1930, Otto Warburg first described that rapidly growing malignant cells had much faster glycolytic rates than normal tissues, and this is referred to as the Warburg effect^49^. Therefore, rare glycolytic mutations are found because cancer cells need to hijack this important pathway to sustain their proliferation and survival. Moreover, recent data has shown that activation of PGI induced by HIF-1 resulted in increased switch of glucose 6-phosphate to fructose 6-phosphate, contributing to cell motility and invasion during cancer metastasis^50^. Consistent with erythrose 4-phosphate, we conjectured the importance of β-D-fructose 6-phosphate in the metabolic process of NPC cells, and targeting it for cancer therapy might be appealing.

### Flavin adenine dinucleotide

The betweenness and permutation FDR of flavin adenine dinucleotide (PubChem ID: CID000643975) were 158 and 0.022, respectively (row 7 of Table 4). Flavin adenine dinucleotide (FAD) is an oxidation-reduction (redox) cofactor involved in the oxidation phosphorylation reaction of cellular metabolism, which can be determined by the ratio of NADH/FAD. As mentioned before, metabolic abnormalities have become an important hallmark of carcinogenesis, and cancerous cells often shift from oxidative phosphorylation to aerobic glycolysis in an attempt to generate ATP for energy^51^,^52^. Unlike in normal cells, reduced oxidative phosphorylation capacity has been observed in highly aggressive cancer cells^52^. Recent findings have also suggested decreased activities and expression of mitochondrial ATP synthases in cancer cells^53^. Given the importance of the electron acceptor FAD for oxidative phosphorylation, we assumed that reactivation of FAD by pharmacologic measures in NPC cells might efficiently suppress disordered metabolism and malignant growth.

### Analysis of enriched KEGG pathways and GO terms of shortest path genes

To further indicate the utility of the NPC-RGCP method, we used a popular analysis tool, DAVID (Database for Annotation, Visualization and Integrated Discovery)^54^, to analyze the putative genes. This tool can help investigators understand biological meaning behind large list of genes. However, there are only five genes remaining. Thus, we selected shortest path genes with FDRs less than 0.05 as the input of the DAVID. The analysis results contained two parts, one is for KEGG pathways and the other is for GO terms, which are available in Supplementary Material IV.

**Figure 3:**
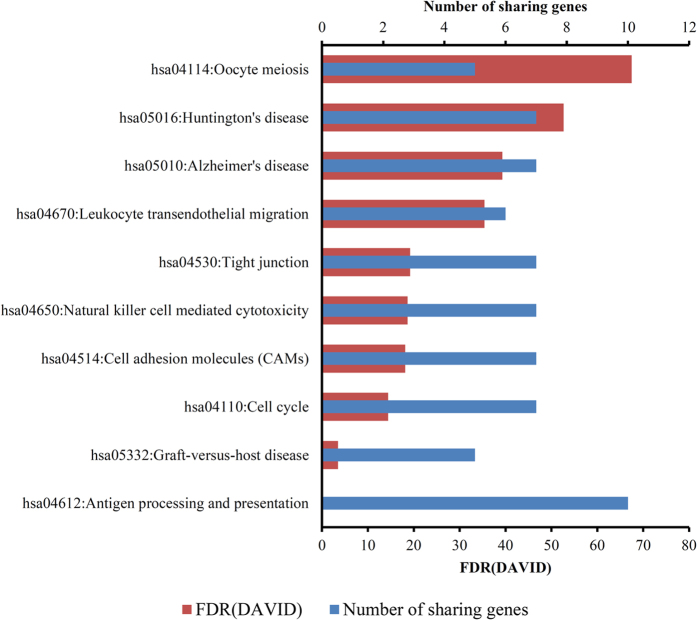
A bar chart illustrating the number of genes among the shortest path genes with FDRs less than 0.05 enriching each KEGG pathway and the FDR obtained by DAVID.

Ten KEGG pathways were found to be enriched by the shortest path genes with FDRs less than 0.05. Figure 3 shows the number of genes among these genes enriching each of ten KEGG pathways and the FDR (which is obtained by DAVID and is different from the FDR calculated using Eq. 2).

Among the ten KEGG pathways, only one pathway, hsa04612: Antigen processing and presentation, had an FDR (DAVID) smaller than 0.05. Ten shortest path genes shared this pathway, indicating the substantial contribution of this pathway to carcinogenesis. As EBV shows strong etiological associations with NPC, its infection has been highly documented to be involved in the multifactorial development of cancer. EBV is a cancerigenic human gamma herpes virus that persistently infects over 90% of adults worldwide. Normally, the EBV viral antigens expressed by the NPC cells are processed and presented by APCs, such as DCs and T-lymphocytes, which triggers an organic adaptive immune response^55^. However, compelling evidence suggests that variable strategies of immune evasion have been evolved by EBV to successfully replicate in infected cells, of which the most important step is inference in the antigen processing machinery and the expression of the major histocompatibility complex (MHC) molecule by viral gene products^56^. For example, BNLF2a can prevent peptide loading of MHC class I molecules by inhibiting the antigen processing-associated transporter, which leads to reduced presentation of viral antigens. Viral BILF1 can reduce the levels of cell surface MHC class I molecules. BGLF5 has been reported to block the synthesis of new MHC class I molecules and regulate the expression of MHC class II molecules. In addition, several viral lytic proteins have been found to hamper the action of effector T cells. Altogether, this significantly enriched pathway demonstrates its close correlations with the susceptibility and aggravation of NPC, expanding new avenues of research as well as providing attractive targets for immunotherapy.

According to Supplementary Material IV, 321 GO terms were identified by DAVID, which are shared by the shortest path genes with FDRs less than 0.05. These GO terms were sorted by the FDRs in increasing order.

It can be observed from Supplementary Material IV that there are three significantly enriched GO terms with FDR (DAVID) smaller than 0.05, which are listed in Table 5. They all belong to biological process (BP) GO terms. Consistent with the previously mentioned enriched KEGG pathways, antigen processing and presentation machinery represents a crucial position in NPC progression. To continue generating viral progeny, EBV learns to manipulate the immune recognition system in host cells. This includes the down-regulation of the most immunogenic latent proteins and restricted expression of some essential genes such as EBNA1 and LMP2. EBNA1 plays pivotal roles in maintaining the EBV episome in the dividing cells, and the presence of a Gly-Ala repeat domain in its sequence prevents it from being presented to MHC class I molecules, thus escaping the recognition of CD8 T cells^57^.

Notably, negative regulation of the protein metabolic process is highly related to NPC. An increasing number of studies have revealed a connection between carcinomas and metabolic syndromes because tumor cells have to survive in nutrient and oxygen poor microenvironments by altering their metabolic pathways. Therefore, many genes involved in metabolism may be attractive targets for clinical diagnosis and therapy. Recently, a polyphenolic flavonoid compound quercetin has been reported to decrease the expression of FASN, which produces a 16-carbon saturated fatty acid and participates in fatty acid synthesis. It also inhibits cell proliferation in NPC cells, suggesting potential associations between lipid metabolism and cell growth. It also indicates that tumor cell growth may be arrested by metabolism-related treatment. Previous studies have shown elevated expression of PTHLH in several NPC cell lines, which has been suggested to regulate the Wnt signaling pathway^58^,^59^. Also, the member Wnt10b in this pathway can increase the sensitivity of cells to insulin, again implying the importance of regulation of metabolic processes in the development of NPC. Moreover, NOR1 shows high levels in the upper respiratory tract including the nasopharynx, but exhibits reduced levels in NPC biopsies and cell lines. Further investigations have revealed that it can alter mitochondrial Bax-Bcl2 balance to suppress tumor cell adaptation to hypoxia.

## Conclusions

This study investigated nasopharyngeal cancer. A network-based method, NPC-RGCP, was constructed to identify novel genes and chemicals related to nasopharyngeal cancer. The analyses indicate that the obtained new putative genes or chemicals may be potential markers, new important therapeutic targets or potential drugs for nasopharyngeal cancer. Furthermore, in view of some studies on disease-related microRNA^60^,^61^, we will focus on integrating the information of microRNA into our network in future, thereby giving an extensive investigation on nasopharyngeal cancer and other diseases.

## Additional Information

**How to cite this article**: Li, Z. *et al.* Identifying novel genes and chemicals related to nasopharyngeal cancer in a heterogeneous network. *Sci. Rep.* **6**, 25515; doi: 10.1038/srep25515 (2016).

## Supplementary Material

Supplementary Information

## Figures and Tables

**Table 1:** Eight NPC-related chemicals retrieved from CTD.

PubChem ID	Chemical name
CID000000264	Butyric acid
CID000000712	Formaldehyde
CID000003385	5-Fluorouracil
CID000006037	Folic acid
CID000006468	PHENCYCLIDINE
CID000148123	Docetaxel trihydrate
CID000445154	Resveratrol
CID009562060	1-Methyl-3-nitro-1-nitrosoguanidine

**Table 2:** The pseudocode of the algorithm for NPC-RGCP.

**Algorithm** NPC-RGCP
**Input:** An NPC-related gene set *S*_*p*_ and an NPC-related chemical set *S*_*c*_, a heterogeneous network
**Output:** A number of putative NPC-related genes and chemicals
1. Searching stage
1.1 Search all shortest paths connecting any two NPC-related genes or chemicals
1.2 Extract inner nodes in these paths; their corresponding genes and chemicals that are not in 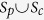 were selected as the shortest path genes or chemicals
2. Screening stage
2.1 For each shortest path gene or chemical, calculate its FDR using Eq. 2
2.2 For each shortest path gene, calculate its MIS and MAS using Eqs 3 and 4, respectively
2.3 For each shortest path chemical, calculate its MIS using Eq. 5
2.4 Select shortest path genes with FDR smaller than 0.05, MIS no less than 900 and MAS no less than 90
2.5 Select shortest path chemicals with FDR smaller than 0.05 and MIS no less than 900
3. Output the remaining shortest path genes and chemicals as the putative NPC-related genes and chemicals

**Table 3:** The detailed information of five putative genes.

Ensembl ID	Gene symbol	Betweenness	Permutation FDR	MIS^a^	MAS^b^
ENSP00000362795	CXCR3	136	0.015	999	139
ENSP00000245414	IRF1	151	0.013	999	114
ENSP00000306043	CDK1	667	0.021	994	233
ENSP00000381607	GSTP1	131	0.019	985	100
ENSP00000269141	CDH2	421	0.03	965	1091

^a^Maximum interaction score.

^b^Maximum alignment score.

**Table 4:** The detailed information of seven putative chemicals.

PubChem ID	Chemical name	Betweenness	Permutation FDR	MIS^a^
CID000023925	Iron	658	0.002	994
CID000001032	Propionic acid	139	<0.001	987
CID000000679	Dimethyl sulfoxide	14	0.048	970
CID000003776	Isopropanol	108	0.013	965
CID000122357	Erythrose 4-phosphate	2	0.024	906
CID000440641	β-D-Fructose 6-phosphate	139	0.001	905
CID000643975	Flavin adenine dinucleotide	158	0.022	900

^a^Maximum interaction score.

**Table 5:** Three enriched GO terms of the shortest path genes.

GO term	Description	Number of sharing genes	FDR
GO:0002474	Antigen processing and presentation of peptide antigen via MHC class I	5	0.014
GO:0032269	Negative regulation of cellular protein metabolic process	10	0.022
GO:0051248	Negative regulation of protein metabolic process	10	0.030
