# Supplementary material for: Identifying novel genes and chemicals related to nasopharyngeal cancer in a heterogeneous network
Source: Sci Rep. 2016 May 5;6:25515. doi: 10.1038/srep25515 (PMC4857740; doi:10.1038/srep25515)
Supplement: Supplementary Information [file srep25515-s1.pdf]

Identifying novel genes and chemicals related to nasopharyngeal cancer in a heterogeneous network

Zhandong Li, Lifeng An, Hao Li, ShaoPeng Wang, You Zhou, Fei Yuan, Lin Li

Supplementary Material I. 132 Ensembl IDs and gene symbols of NPC-related genes and their sources

Gene symbol	Ensembl ID	Source
STAT1	ENSP00000354394	NCI
PIK3CA	ENSP00000263967	UniProt
CXCR4	ENSP00000386884	NCI
PPP1R11	ENSP00000411038	NCI
BPIFA1	ENSP00000346251	UniProt
HLA-A	ENSP00000416233	NCI
HLA-E	ENSP00000397420	NCI
RAC1	ENSP00000348461	NCI
PPP1R11	ENSP00000403557	NCI
HLA-A	ENSP00000388526	NCI
TAP1	ENSP00000402316	NCI
ADAMTS9	ENSP00000418735	TSGenes
NOTCH1	ENSP00000277541	NCI

MRPL47	ENSP00000417602	UniProt
THY1	ENSP00000284240	NCI, TSGenes
XRCC1	ENSP00000262887	NCI
HLA-E	ENSP00000402694	NCI
HLA-B	ENSP00000399168	NCI
PPP1R11	ENSP00000412297	NCI
TAP1	ENSP00000401149	NCI
PPP1R10	ENSP00000407310	NCI
FSCN1	ENSP00000371798	NCI
TAP1	ENSP00000412933	NCI
AFP	ENSP00000226359	NCI
PPP1R10	ENSP00000407181	NCI
HLA-E	ENSP00000409910	NCI
BDNF	ENSP00000414303	NCI
PCDH10	ENSP00000264360	NCI, TSGenes
DNMT1	ENSP00000352516	NCI
HLA-A	ENSP00000398188	NCI
BRD7	ENSP00000378180	NCI
PPP1R10	ENSP00000389299	NCI
PPP1R11	ENSP00000394056	NCI

HLA-A	ENSP00000373114	NCI
HLA-A	ENSP00000388724	NCI
HMGB1	ENSP00000343040	NCI
CADM1	ENSP00000329797	TSGenes
MIF	ENSP00000215754	NCI
PPP1R11	ENSP00000373107	NCI
CHFR	ENSP00000320557	NCI
MYL12B	ENSP00000237500	UniProt
CD44	ENSP00000278385	NCI
NEDD4	ENSP00000345530	NCI
TKTL2	ENSP00000280605	NCI
GSTT1	ENSP00000248935	NCI
GSTT1	ENSP00000401632	NCI
PPP1R10	ENSP00000416060	NCI
LIG3	ENSP00000367787	NCI
EDNRB	ENSP00000335311	NCI
PPP1R11	ENSP00000414808	NCI
ICAM1	ENSP00000264832	NCI
VWA5A	ENSP00000376504	TSGenes
BRD3	ENSP00000305918	NCI

CXCL10	ENSP00000305651	NCI
HLA-E	ENSP00000373091	NCI
HLA-E	ENSP00000390707	NCI
HLA-A	ENSP00000410645	NCI
SRC	ENSP00000350941	NCI
PPP1R11	ENSP00000407981	NCI
PPP1R10	ENSP00000373080	NCI
TAP1	ENSP00000415660	NCI
DLC1	ENSP00000276297	NCI, TSGenes
TAP1	ENSP00000413080	NCI
LRRC4	ENSP00000249363	UniProt
ARHGAP44	ENSP00000368994	UniProt
TKT	ENSP00000405455	NCI
COX7B2	ENSP00000305964	UniProt, NCI
PPP1R10	ENSP00000413554	NCI
KLF6	ENSP00000419923	NCI
CCR7	ENSP00000246657	NCI
ANG	ENSP00000336762	NCI
NICN1	ENSP00000273598	UniProt
CCDC136	ENSP00000297788	UniProt

CDH4	ENSP00000353656	TSGenes
TFPI2	ENSP00000222543	TSGenes
STC1	ENSP00000290271	NCI
TAP1	ENSP00000372722	NCI
MGMT	ENSP00000302111	NCI
ETS1	ENSP00000376436	NCI
CDK4	ENSP00000257904	NCI
TMEM8B	ENSP00000367227	UniProt
HLA-A	ENSP00000366005	NCI
RECK	ENSP00000367202	NCI
NAT2	ENSP00000286479	NCI
TAP1	ENSP00000346206	NCI
CBWD1	ENSP00000348915	UniProt
PTEN	ENSP00000361021	NCI, TSGenes
NQO1	ENSP00000319788	NCI
CCND1	ENSP00000227507	NCI
CD70	ENSP00000245903	NCI
HSBP1	ENSP00000392896	UniProt
TRAF1	ENSP00000362994	NCI
PPP1R11	ENSP00000365963	NCI

GADD45G	ENSP00000252506	TSGenes
SYK	ENSP00000364898	NCI
FBXO30	ENSP00000237281	UniProt, NCI
FAS	ENSP00000347979	NCI
OPCML	ENSP00000330862	TSGenes
HLA-B	ENSP00000400842	NCI
SDHD	ENSP00000364699	NCI
HLA-E	ENSP00000365817	NCI
CD40	ENSP00000361359	NCI
RUNX3	ENSP00000343477	NCI
BAX	ENSP00000293288	NCI
RASSF1	ENSP00000349547	TSGenes
LTF	ENSP00000231751	NCI, TSGenes
IFNA17	ENSP00000411940	NCI
PPP1R10	ENSP00000365694	NCI
ZMYND10	ENSP00000231749	TSGenes
C9orf53	ENSP00000414893	UniProt
CYP2E1	ENSP00000252945	NCI
STAT3	ENSP00000264657	NCI
CYP1A1	ENSP00000369050	NCI

GSTM1	ENSP00000311469	NCI
N4BP2	ENSP00000261435	NCI
VHL	ENSP00000256474	NCI
IRF8	ENSP00000268638	TSGenes
TP53	ENSP00000269305	UniProt
CD58	ENSP00000358501	NCI
PRR4	ENSP00000228811	UniProt
ITIH4	ENSP00000266041	NCI
OSCP1	ENSP00000349052	UniProt
DUSP6	ENSP00000279488	TSGenes
MDM2	ENSP00000417281	NCI
PIGR	ENSP00000348888	NCI
TLR3	ENSP00000296795	NCI
CR2	ENSP00000356024	NCI
TAP1	ENSP00000405356	NCI
FHIT	ENSP00000342087	NCI, TSGenes
EZR	ENSP00000338934	UniProt
UBAP1	ENSP00000297661	UniProt, NCI
KIAA1045	ENSP00000242315	UniProt

Supplementary Material II. Shortest path genes and chemicals obtained by the searching stage of the NPC-RGCP method

1. 325 shortest path genes

Ensembl ID	Gene symbol	Betweenness
ENSP00000344818	UBC	3270
ENSP00000340858	B2M	1666
ENSP00000003084	CFTR	1250
ENSP00000206249	ESR1	839
ENSP00000335153	HSP90AA1	797
ENSP00000256442	CCNB1	793
ENSP00000287598	BUB1B	772
ENSP00000352064	KLRC1	685
ENSP00000338130	KLRD1	670
ENSP00000306043	CDK1	667
ENSP00000264033	CBL	662
ENSP00000344456	CTNNB1	628
ENSP00000353224	TFRC	610
ENSP00000417404	HFE	610
ENSP00000338018	HIF1A	567
ENSP00000360266	JUN	551
ENSP00000326031	PPP1CA	549

ENSP00000359890	MBNL3	544
ENSP00000363055	ZWINT	544
ENSP00000368438	PCNA	531
ENSP00000309845	HRAS	495
ENSP00000235090	WDR77	467
ENSP00000357858	BUB3	459
ENSP00000378165	ZNF207	459
ENSP00000311677	PPP1R8	454
ENSP00000262613	SLC9A3R1	450
ENSP00000269141	CDH2	421
ENSP00000355759	PARP1	413
ENSP00000274026	CCNA2	398
ENSP00000262367	CREBBP	397
ENSP00000378546	TAPBP	371
ENSP00000320866	CALR	369
ENSP00000275493	EGFR	352
ENSP00000172229	NGFR	336
ENSP00000300093	PLK1	307
ENSP00000339109	ANAPC1	284
ENSP00000234038	PPP1R7	276

ENSP00000247668	TRAF2	276
ENSP00000313950	AURKB	275
ENSP00000363921	PARD3	274
ENSP00000219255	PARD6A	271
ENSP00000303242	ITGB2	270
ENSP00000251849	RAF1	268
ENSP00000247461	CANX	265
ENSP00000300289	PDIA3	265
ENSP00000315644	TYMS	243
ENSP00000339007	GRB2	239
ENSP00000267163	RB1	226
ENSP00000270202	AKT1	223
ENSP00000364094	ITGB1	200
ENSP00000367316	ITGA8	200
ENSP00000346839	FN1	195
ENSP00000401303	SHC1	188
ENSP00000351486	NTRK1	185
ENSP00000293379	ITGA5	178
ENSP00000263253	EP300	176
ENSP00000215832	MAPK1	171

ENSP00000268035	IGF1R	161
ENSP00000308450	CDC20	160
ENSP00000314458	CDC42	154
ENSP00000245414	IRF1	151
ENSP00000262643	CCNE1	148
ENSP00000418447	PPP2CA	148
ENSP00000292644	PSMC2	146
ENSP00000281708	FBXW7	143
ENSP00000342215	KIR2DL3	142
ENSP00000362649	HDAC1	142
ENSP00000407195	TAPBP	141
ENSP00000010338	TRAF3IP3	139
ENSP00000164227	BCL3	139
ENSP00000221494	SF3A2	139
ENSP00000221972	CD79A	139
ENSP00000227758	BIRC2	139
ENSP00000232375	PFKFB4	139
ENSP00000248244	TICAM1	139
ENSP00000254231	LIN28A	139
ENSP00000260102	MRPL15	139

ENSP00000264010	CTCF	139
ENSP00000265132	AMBP	139
ENSP00000266079	PRPF6	139
ENSP00000266557	CD27	139
ENSP00000287497	ITGAM	139
ENSP00000291527	TFF1	139
ENSP00000292123	SAFB	139
ENSP00000299106	JAM3	139
ENSP00000302021	MUC7	139
ENSP00000302961	HSPA4	139
ENSP00000304669	CTNNA1	139
ENSP00000304903	CD2BP2	139
ENSP00000311344	PPP2R1B	139
ENSP00000315997	LILRB1	139
ENSP00000316578	SUZ12	139
ENSP00000320147	EZH2	139
ENSP00000321259	TALDO1	139
ENSP00000329292	GKN2	139
ENSP00000338983	MUC1	139
ENSP00000344352	ATF3	139

ENSP00000346022	RPL9	139
ENSP00000350720	SMARCA4	139
ENSP00000358490	CD2	139
ENSP00000359345	RPL5	139
ENSP00000361125	VEGFA	139
ENSP00000361405	MMP9	139
ENSP00000361813	BEX1	139
ENSP00000368683	EDN1	139
ENSP00000369519	MTAP	139
ENSP00000378288	MYLK3	139
ENSP00000386759	SETD2	139
ENSP00000397552	ACTL6A	139
ENSP00000043402	RTN4R	138
ENSP00000224337	BLNK	138
ENSP00000266000	DAXX	138
ENSP00000337838	RTN4	138
ENSP00000355778	H3F3A	138
ENSP00000362441	ATRX	138
ENSP00000384442	CDK11A	138
ENSP00000262629	TYROBP	137

ENSP00000265056	MCM2	137
ENSP00000295926	CCNL1	137
ENSP00000313419	CD19	137
ENSP00000367934	UQCRQ	137
ENSP00000263753	SGOL1	136
ENSP00000295797	PRKCI	136
ENSP00000329967	TBK1	136
ENSP00000354901	CXCL9	136
ENSP00000362795	CXCR3	136
ENSP00000363998	ITCH	136
ENSP00000374455	SQSTM1	136
ENSP00000044462	PSMA4	135
ENSP00000308541	F2	135
ENSP00000337915	CYP3A4	135
ENSP00000406797	PSMB8	135
ENSP00000338297	IGF2	134
ENSP00000355325	PSMB5	134
ENSP00000371634	IGF2BP2	134
ENSP00000268058	PML	133
ENSP00000247655	COX7C	131

ENSP00000302269	VAV1	131
ENSP00000354499	MT-CO1	131
ENSP00000354876	MT-CO2	131
ENSP00000381607	GSTP1	131
ENSP00000242057	AHR	130
ENSP00000306245	FOS	129
ENSP00000358525	NGF	126
ENSP00000351407	ARNT	124
ENSP00000219548	STUB1	115
ENSP00000307863	U2AF2	113
ENSP00000365439	HNRNPK	113
ENSP00000189444	NFKB2	109
ENSP00000396308	DHFR	105
ENSP00000011653	CD4	103
ENSP00000337825	LCK	103
ENSP00000302530	BUB1	100
ENSP00000223129	RPA3	98
ENSP00000254719	RPA1	98
ENSP00000284384	PRKCA	98
ENSP00000301838	FADD	96

ENSP00000259808	RIPK1	95
ENSP00000313829	KHDRBS1	92
ENSP00000261461	PPP2R5A	89
ENSP00000262320	AXIN1	89
ENSP00000266970	CDK2	88
ENSP00000358022	MCL1	88
ENSP00000363832	AOX1	86
ENSP00000356438	PTGS2	85
ENSP00000384675	SOS1	81
ENSP00000311032	CASP3	80
ENSP00000264554	SHC2	77
ENSP00000277120	NTRK2	77
ENSP00000304895	IRS1	77
ENSP00000360916	VAV2	77
ENSP00000309503	YWHAZ	72
ENSP00000277010	SIGMAR1	71
ENSP00000249071	RAC2	70
ENSP00000329380	GP1BA	69
ENSP00000357656	FYN	68
ENSP00000279593	GRIN2B	66

ENSP00000355155	GRIN3A	66
ENSP00000360616	GRIN1	66
ENSP00000274335	PIK3R1	61
ENSP00000302486	MAP2K1	61
ENSP00000286827	TIAM1	58
ENSP00000260433	CYP19A1	54
ENSP00000363822	AR	54
ENSP00000228945	ARHGDIB	49
ENSP00000354859	DRD2	44
ENSP00000351273	CASP8	43
ENSP00000310127	IRF3	41
ENSP00000231487	SKP1	39
ENSP00000375608	KIR3DL1	39
ENSP00000278379	SLC1A2	37
ENSP00000407431	HLA-C	36
ENSP00000353059	APAF1	35
ENSP00000223023	WASL	34
ENSP00000311113	JUP	34
ENSP00000341189	PTK2	34
ENSP00000308938	PLG	32

ENSP00000320940	NCOA1	32
ENSP00000226574	NFKB1	30
ENSP00000229854	MCM3	30
ENSP00000370473	IGFBP3	30
ENSP00000244741	CDKN1A	29
ENSP00000302665	IGF1	29
ENSP00000302955	RRM2	28
ENSP00000242577	DYNLL1	26
ENSP00000303830	INSR	26
ENSP00000348986	INS-IGF2	26
ENSP00000359206	BTRC	26
ENSP00000219476	TSC2	25
ENSP00000245960	CDC25B	25
ENSP00000269321	ARHGDIA	24
ENSP00000269571	ERBB2	24
ENSP00000371067	JAK2	24
ENSP00000384273	RELA	24
ENSP00000215829	SNRPD3	23
ENSP00000332643	NDN	22
ENSP00000345571	E2F1	22

ENSP00000227378	HSPA8	21
ENSP00000254066	RARA	21
ENSP00000263309	CLNS1A	21
ENSP00000314491	SRRT	21
ENSP00000319169	PRMT5	21
ENSP00000329623	BCL2	21
ENSP00000409132	HLA-G	21
ENSP00000228307	PXN	20
ENSP00000380227	ITGA4	20
ENSP00000216225	RBX1	19
ENSP00000287820	PPARG	19
ENSP00000326366	PSEN1	19
ENSP00000367207	MYC	19
ENSP00000324804	PPP2R1A	18
ENSP00000382004	CTNND1	18
ENSP00000278568	PAK1	17
ENSP00000288986	NCK1	17
ENSP00000046794	LCP2	15
ENSP00000209728	CDC6	15
ENSP00000357177	ARHGEF11	15

ENSP00000400175	RHOA	15
ENSP00000248566	SHFM1	13
ENSP00000282561	GJA1	13
ENSP00000296223	POLR2H	13
ENSP00000300161	YWHAB	13
ENSP00000300574	CRK	13
ENSP00000312735	POLR2B	13
ENSP00000315325	ARHGEF2	13
ENSP00000222005	CDC37	12
ENSP00000287936	HMGCR	12
ENSP00000297494	NOS3	12
ENSP00000342952	ADCY2	12
ENSP00000162330	BCAR1	11
ENSP00000324806	GSK3B	11
ENSP00000356087	IKBKE	11
ENSP00000358541	SIKE1	11
ENSP00000268182	IQGAP1	10
ENSP00000309103	BAD	10
ENSP00000261597	NDC80	8
ENSP00000295897	ALB	8

ENSP00000351908	MAP3K5	8
ENSP00000365891	WAS	8
ENSP00000329357	SP1	7
ENSP00000361423	ABL1	7
ENSP00000252102	NDUFA2	6
ENSP00000252622	LSM7	6
ENSP00000264156	MCM6	6
ENSP00000275766	ZC3HAV1L	6
ENSP00000301019	CDT1	6
ENSP00000315702	MOB4	6
ENSP00000361626	YBX1	6
ENSP00000398597	EXOSC6	6
ENSP00000228872	CDKN1B	5
ENSP00000257068	MTNR1B	5
ENSP00000261769	CDH1	5
ENSP00000274255	SKP2	5
ENSP00000302564	BCL2L1	5
ENSP00000376076	SUMO1	5
ENSP00000391592	PTPN6	5
ENSP00000252029	TYMP	4

ENSP00000263734	EPAS1	4
ENSP00000278616	ATM	4
ENSP00000363641	TXN	4
ENSP00000381331	HDAC2	4
ENSP00000162749	TNFRSF1A	3
ENSP00000243077	LRP1	3
ENSP00000292303	CCR5	3
ENSP00000293272	CCL5	3
ENSP00000307288	MCM7	3
ENSP00000323929	A2M	3
ENSP00000360683	PTPN1	3
ENSP00000223095	SERPINE1	2
ENSP00000226218	SEBOX	2
ENSP00000232458	ECT2	2
ENSP00000245907	C3	2
ENSP00000259075	TANK	2
ENSP00000260363	KIF23	2
ENSP00000261023	ITGAV	2
ENSP00000268296	ITGAX	2
ENSP00000281537	TJP1	2

ENSP00000289779	F11R	2
ENSP00000306397	UQCRFS1	2
ENSP00000324648	CYP2B6	2
ENSP00000324729	SAV1	2
ENSP00000343204	JAK1	2
ENSP00000361892	STK4	2
ENSP00000364649	SDHB	2
ENSP00000368572	SAT1	2
ENSP00000390500	STK3	2
ENSP00000203407	UQCRC1	1
ENSP00000223321	PSMA2	1
ENSP00000254810	H3F3B	1
ENSP00000258962	SRSF1	1
ENSP00000260682	CYP2C9	1
ENSP00000264708	POMC	1
ENSP00000303706	CDC25A	1
ENSP00000308815	CCL19	1
ENSP00000309565	UQCRH	1
ENSP00000315859	RNPS1	1
ENSP00000317159	CYC1	1

ENSP00000332468	TRAF3	1
ENSP00000337773	NQO2	1
ENSP00000354554	MT-CYB	1
ENSP00000354558	MTOR	1
ENSP00000354961	MT-ND4	1
ENSP00000358617	PHTF1	1
ENSP00000359998	GSTA4	1
ENSP00000363827	HSPG2	1

2. 64 shortest path chemicals

PubChem ID	Chemical name	Betweenness
CID000005957	adenosine triphosphate	2157
CID000000271	calcium	1692
CID000023994	zinc	804
CID000023925	Iron	658
CID000006830	guanosine triphosphate	433
CID000000888	magnesium	367
CID000000977	oxygen	356
CID000000753	glycerol	347
CID000001132	thiamine pyrophosphate	276

CID000087642	coenzyme A	192
CID000033032	glutamic acid	172
CID000643975	flavin adenine dinucleotide	158
CID000006083	adenosine monophosphate	150
CID000000311	citric acid	142
CID000006022	adenosine diphosphate	141
CID000001032	Propionic acid	139
CID000440641	β-D-Fructose 6-phosphate	139
CID005280360	PGE2	138
CID000001005	phosphoenolpyruvate	136
CID000005961	glutamine	136
CID000124886	Glutathione	136
CID000000177	acetaldehyde	135
CID000000923	sodium	135
CID000000979	p-hydroxyphenylpyruvate	135
CID000439153	reduced nicotinamide adenine dinucleotide	135
CID000000312	chloride	131
CID000444493	acetyl-CoA	131
CID002733525	tamoxifen	119
CID000004055	menadione	117

CID000005892	beta-NAD	115
CID000003776	isopropanol	108
CID000023930	manganese	90
CID000000283	formate	87
CID000005035	raloxifene	86
CID000439176	5'-methylthioadenosine	78
CID000001102	spermidine	76
CID000003559	haloperidol	71
CID000000190	adenine	62
CID000006013	testosterone	54
CID000000681	dopamine	44
CID000005885	NADP	34
CID000000961	hydroxyl radicals	32
CID000003715	indomethacin	27
CID000439155	AdoHcy	25
CID000444899	arachidonic acid	24
CID000000679	Dimethyl sulfoxide	14
CID000000051	alpha-ketoglutarate	13
CID000006322	arginine	12
CID000000222	ammonia	5

CID000000896	melatonin	5
CID000008977	guanosine diphosphate	4
CID000065063	dUMP	4
CID000005789	thymidine	3
CID000000750	glycine	2
CID000001103	spermine	2
CID000004763	phenobarbital	2
CID000005950	L-alanine	2
CID000122357	erythrose 4-phosphate	2
CID000001003	phosphate	1
CID000001135	thymine	1
CID000002733	chlorzoxazone	1
CID000003032	diclofenac	1
CID000004174	metyrapone	1
CID000145742	L-proline	1

Supplementary Material III. The permutation FDRs, MIS, and MAS of the candidate genes and chemicals obtained by the searching stage of the NPC-RGCP method

1. 325 shortest path genes

Ensembl ID	Gene symbol	Betweenness	Fermutation FDR	MIS	MAS
ENSP00000164227	BCL3	139	<0.001	941	65
ENSP00000232375	PFKFB4	139	<0.001	899	25
ENSP00000234038	PPP1R7	276	<0.001	619	45
ENSP00000254231	LIN28A	139	<0.001	507	21
ENSP00000260102	MRPL15	139	<0.001	853	21
ENSP00000264010	CTCF	139	<0.001	800	59
ENSP00000265132	AMBP	139	<0.001	899	62
ENSP00000266557	CD27	139	<0.001	997	57
ENSP00000275766	ZC3HAV1L	6	<0.001	266	0
ENSP00000287598	BUB1B	772	<0.001	566	26
ENSP00000291527	TFF1	139	<0.001	677	20
ENSP00000292123	SAFB	139	<0.001	644	26
ENSP00000295926	CCNL1	137	<0.001	937	24
ENSP00000299106	JAM3	139	<0.001	839	45
ENSP00000311677	PPP1R8	454	<0.001	306	21
ENSP00000315997	LILRB1	139	<0.001	953	23
ENSP00000321259	TALDO1	139	<0.001	995	22

ENSP00000326031	PPP1CA	549	<0.001	972	23
ENSP00000329292	GKN2	139	<0.001	893	23
ENSP00000337773	NQO2	1	<0.001	781	214
ENSP00000338130	KLRD1	670	<0.001	990	25
ENSP00000340858	B2M	1666	<0.001	999	44
ENSP00000352064	KLRC1	685	<0.001	996	22
ENSP00000353224	TFRC	610	<0.001	906	24
ENSP00000355759	PARP1	413	<0.001	999	50
ENSP00000359890	MBNL3	544	<0.001	202	27
ENSP00000361813	BEX1	139	<0.001	808	21
ENSP00000363055	ZWINT	544	<0.001	380	21
ENSP00000369519	MTAP	139	<0.001	463	22
ENSP00000375608	KIR3DL1	39	<0.001	988	23
ENSP00000378288	MYLK3	139	<0.001	992	84
ENSP00000378546	TAPBP	371	<0.001	996	22
ENSP00000386759	SETD2	139	<0.001	931	28
ENSP00000407195	TAPBP	141	<0.001	939	22
ENSP00000417404	HFE	610	<0.001	563	176
ENSP00000221972	CD79A	139	0.001	981	29
ENSP00000277010	SIGMAR1	71	0.001	218	23

ENSP00000300289	PDIA3	265	0.001	965	24
ENSP00000302021	MUC7	139	0.001	719	0
ENSP00000316578	SUZ12	139	0.001	744	0
ENSP00000320866	CALR	369	0.001	956	22
ENSP00000358617	PHTF1	1	0.001	899	0
ENSP00000010338	TRAF3IP3	139	0.002	999	0
ENSP00000311344	PPP2R1B	139	0.002	800	26
ENSP00000355155	GRIN3A	66	0.002	291	27
ENSP00000358490	CD2	139	0.002	999	27
ENSP00000247461	CANX	265	0.003	939	0
ENSP00000266079	PRPF6	139	0.003	182	23
ENSP00000304903	CD2BP2	139	0.003	845	27
ENSP00000363921	PARD3	274	0.003	899	27
ENSP00000368683	EDN1	139	0.003	998	21
ENSP00000172229	NGFR	336	0.004	996	49
ENSP00000248244	TICAM1	139	0.004	999	28
ENSP00000262613	SLC9A3R1	450	0.004	999	22
ENSP00000203407	UQCRC1	1	0.006	941	0
ENSP00000219255	PARD6A	271	0.006	992	0
ENSP00000368572	SAT1	2	0.006	425	20

ENSP00000378165	ZNF207	459	0.006	0	23
ENSP00000235090	WDR77	467	0.007	697	24
ENSP00000265056	MCM2	137	0.007	977	24
ENSP00000268296	ITGAX	2	0.007	970	30
ENSP00000281708	FBXW7	143	0.007	999	26
ENSP00000303242	ITGB2	270	0.007	999	29
ENSP00000308815	CCL19	1	0.007	998	20
ENSP00000339109	ANAPC1	284	0.007	0	25
ENSP00000357858	BUB3	459	0.007	368	23
ENSP00000264156	MCM6	6	0.008	705	22
ENSP00000302955	RRM2	28	0.009	732	23
ENSP00000360616	GRIN1	66	0.009	457	23
ENSP00000243077	LRP1	3	0.011	988	72
ENSP00000257068	MTNR1B	5	0.012	899	74
ENSP00000245414	IRF1	151	0.013	999	114
ENSP00000371634	IGF2BP2	134	0.013	611	27
ENSP00000286827	TIAM1	58	0.014	999	25
ENSP00000287497	ITGAM	139	0.014	999	32
ENSP00000262643	CCNE1	148	0.015	999	70
ENSP00000274026	CCNA2	398	0.015	999	86

ENSP00000323929	A2M	3	0.015	924	28
ENSP00000362795	CXCR3	136	0.015	999	139
ENSP00000232458	ECT2	2	0.017	899	31
ENSP00000304669	CTNNA1	139	0.017	976	25
ENSP00000384442	CDK11A	138	0.017	899	211
ENSP00000381607	GSTP1	131	0.019	985	100
ENSP00000189444	NFKB2	109	0.02	914	49
ENSP00000261461	PPP2R5A	89	0.02	720	22
ENSP00000342215	KIR2DL3	142	0.02	925	22
ENSP00000306043	CDK1	667	0.021	994	233
ENSP00000228945	ARHGDIB	49	0.022	970	21
ENSP00000278379	SLC1A2	37	0.022	946	28
ENSP00000337838	RTN4	138	0.023	800	23
ENSP00000397552	ACTL6A	139	0.023	995	26
ENSP00000406797	PSMB8	135	0.023	938	21
ENSP00000320147	EZH2	139	0.024	949	23
ENSP00000363832	AOX1	86	0.024	412	26
ENSP00000295797	PRKCI	136	0.025	965	68
ENSP00000354901	CXCL9	136	0.025	991	55
ENSP00000367934	UQCRQ	137	0.025	919	20

ENSP00000252029	TYMP	4	0.026	620	22
ENSP00000367316	ITGA8	200	0.026	0	26
ENSP00000309565	UQCRH	1	0.028	907	20
ENSP00000361405	MMP9	139	0.028	989	28
ENSP00000269141	CDH2	421	0.03	965	1091
ENSP00000315644	TYMS	243	0.031	611	0
ENSP00000407431	HLA-C	36	0.031	556	556
ENSP00000044462	PSMA4	135	0.032	899	21
ENSP00000332643	NDN	22	0.034	926	21
ENSP00000357177	ARHGEF11	15	0.034	921	25
ENSP00000247655	COX7C	131	0.036	401	22
ENSP00000355325	PSMB5	134	0.036	899	22
ENSP00000043402	RTN4R	138	0.037	490	132
ENSP00000354859	DRD2	44	0.038	899	45
ENSP00000315325	ARHGEF2	13	0.039	997	27
ENSP00000224337	BLNK	138	0.04	999	30
ENSP00000284384	PRKCA	98	0.04	994	83
ENSP00000344352	ATF3	139	0.04	997	26
ENSP00000359998	GSTA4	1	0.042	983	47
ENSP00000362441	ATRX	138	0.042	466	25

ENSP00000296223	POLR2H	13	0.043	899	26
ENSP00000223129	RPA3	98	0.044	917	19
ENSP00000259075	TANK	2	0.044	871	24
ENSP00000338297	IGF2	134	0.045	925	0
ENSP00000263734	EPAS1	4	0.047	999	0
ENSP00000364649	SDHB	2	0.047	998	25
ENSP00000355778	H3F3A	138	0.05	925	25
ENSP00000247668	TRAF2	276	0.051	999	278
ENSP00000302961	HSPA4	139	0.052	993	25
ENSP00000313419	CD19	137	0.053	999	30
ENSP00000254810	H3F3B	1	0.054	925	25
ENSP00000306397	UQCRFS1	2	0.055	959	23
ENSP00000226574	NFKB1	30	0.056	943	42
ENSP00000338983	MUC1	139	0.056	989	0
ENSP00000360266	JUN	551	0.056	994	0
ENSP00000363998	ITCH	136	0.059	997	525
ENSP00000256442	CCNB1	793	0.063	992	62
ENSP00000359206	BTRC	26	0.063	867	27
ENSP00000363827	HSPG2	1	0.063	904	94
ENSP00000363641	TXN	4	0.068	906	22

ENSP00000227758	BIRC2	139	0.07	999	33
ENSP00000277120	NTRK2	77	0.071	999	182
ENSP00000324648	CYP2B6	2	0.079	962	440
ENSP00000264554	SHC2	77	0.081	920	47
ENSP00000302269	VAV1	131	0.085	999	62
ENSP00000351486	NTRK1	185	0.087	967	169
ENSP00000361892	STK4	2	0.088	996	91
ENSP00000266000	DAXX	138	0.089	993	25
ENSP00000346022	RPL9	139	0.089	191	21
ENSP00000301838	FADD	96	0.09	999	23
ENSP00000313950	AURKB	275	0.09	927	64
ENSP00000360916	VAV2	77	0.092	989	60
ENSP00000329967	TBK1	136	0.095	900	70
ENSP00000401303	SHC1	188	0.096	963	55
ENSP00000351908	MAP3K5	8	0.097	972	82
ENSP00000262367	CREBBP	397	0.101	999	79
ENSP00000275493	EGFR	352	0.102	999	199
ENSP00000324729	SAV1	2	0.103	956	53
ENSP00000368438	PCNA	531	0.103	999	22
ENSP00000361125	VEGFA	139	0.107	994	24

ENSP00000338018	HIF1A	567	0.112	999	25
ENSP00000254719	RPA1	98	0.117	999	0
ENSP00000301019	CDT1	6	0.117	952	24
ENSP00000396308	DHFR	105	0.117	783	22
ENSP00000293272	CCL5	3	0.119	994	23
ENSP00000350720	SMARCA4	139	0.119	996	49
ENSP00000356087	IKBKE	11	0.127	855	81
ENSP00000358541	SIKE1	11	0.128	899	23
ENSP00000390500	STK3	2	0.128	985	89
ENSP00000351273	CASP8	43	0.134	999	23
ENSP00000242057	AHR	130	0.135	980	24
ENSP00000260682	CYP2C9	1	0.142	937	561
ENSP00000303706	CDC25A	1	0.142	929	26
ENSP00000311113	JUP	34	0.145	995	23
ENSP00000268058	PML	133	0.148	999	33
ENSP00000310127	IRF3	41	0.148	914	131
ENSP00000391592	PTPN6	5	0.151	997	43
ENSP00000245907	C3	2	0.155	997	25
ENSP00000229854	MCM3	30	0.161	746	28
ENSP00000317159	CYC1	1	0.172	956	0

ENSP00000215832	MAPK1	171	0.173	999	172
ENSP00000351407	ARNT	124	0.174	915	27
ENSP00000249071	RAC2	70	0.175	946	360
ENSP00000260363	KIF23	2	0.178	350	24
ENSP00000300093	PLK1	307	0.179	920	70
ENSP00000293379	ITGA5	178	0.193	918	27
ENSP00000374455	SQSTM1	136	0.198	837	0
ENSP00000364094	ITGB1	200	0.199	932	43
ENSP00000245960	CDC25B	25	0.201	489	28
ENSP00000260433	CYP19A1	54	0.208	356	94
ENSP00000261597	NDC80	8	0.209	427	27
ENSP00000324804	PPP2R1A	18	0.213	800	25
ENSP00000259808	RIPK1	95	0.215	995	110
ENSP00000308450	CDC20	160	0.217	991	23
ENSP00000400175	RHOA	15	0.22	867	211
ENSP00000358022	MCL1	88	0.228	999	52
ENSP00000344456	CTNNB1	628	0.231	999	25
ENSP00000354876	MT-CO2	131	0.231	400	20
ENSP00000244741	CDKN1A	29	0.232	999	23
ENSP00000356438	PTGS2	85	0.234	984	40

ENSP00000292303	CCR5	3	0.237	978	149
ENSP00000354499	MT-CO1	131	0.24	384	0
ENSP00000222005	CDC37	12	0.246	999	26
ENSP00000359345	RPL5	139	0.25	999	0
ENSP00000380227	ITGA4	20	0.252	932	26
ENSP00000268035	IGF1R	161	0.261	992	189
ENSP00000307288	MCM7	3	0.263	946	27
ENSP00000311032	CASP3	80	0.265	937	23
ENSP00000361626	YBX1	6	0.271	996	0
ENSP00000281537	TJP1	2	0.273	923	26
ENSP00000226218	SEBOX	2	0.275	953	26
ENSP00000219548	STUB1	115	0.28	975	23
ENSP00000263753	SGOL1	136	0.281	0	25
ENSP00000418447	PPP2CA	148	0.283	818	22
ENSP00000358525	NGF	126	0.285	950	133
ENSP00000289779	F11R	2	0.29	914	39
ENSP00000332468	TRAF3	1	0.291	999	237
ENSP00000162749	TNFRSF1A	3	0.296	996	37
ENSP00000223321	PSMA2	1	0.298	906	25
ENSP00000409132	HLA-G	21	0.314	804	532

ENSP00000308541	F2	135	0.316	907	25
ENSP00000345571	E2F1	22	0.317	990	31
ENSP00000353059	APAF1	35	0.333	992	24
ENSP00000262629	TYROBP	137	0.337	999	21
ENSP00000223095	SERPINE1	2	0.345	950	23
ENSP00000003084	CFTR	1250	0.354	960	74
ENSP00000261023	ITGAV	2	0.365	959	27
ENSP00000302564	BCL2L1	5	0.365	999	59
ENSP00000382004	CTNND1	18	0.371	999	27
ENSP00000346839	FN1	195	0.376	998	27
ENSP00000342952	ADCY2	12	0.377	904	27
ENSP00000282561	GJA1	13	0.378	997	25
ENSP00000278568	PAK1	17	0.39	999	99
ENSP00000288986	NCK1	17	0.393	970	74
ENSP00000261769	CDH1	5	0.396	995	548
ENSP00000329380	GP1BA	69	0.398	262	50
ENSP00000337915	CYP3A4	135	0.399	963	101
ENSP00000302486	MAP2K1	61	0.4	933	82
ENSP00000267163	RB1	226	0.404	999	26
ENSP00000309103	BAD	10	0.413	855	22

ENSP00000300161	YWHAB	13	0.414	512	29
ENSP00000046794	LCP2	15	0.417	999	44
ENSP00000279593	GRIN2B	66	0.417	958	28
ENSP00000011653	CD4	103	0.424	995	33
ENSP00000309503	YWHAZ	72	0.436	908	27
ENSP00000269321	ARHGDIA	24	0.439	999	23
ENSP00000231487	SKP1	39	0.446	990	20
ENSP00000343204	JAK1	2	0.45	999	149
ENSP00000242577	DYNLL1	26	0.452	789	22
ENSP00000254066	RARA	21	0.455	990	23
ENSP00000268182	IQGAP1	10	0.466	999	24
ENSP00000295897	ALB	8	0.475	927	496
ENSP00000381331	HDAC2	4	0.485	999	22
ENSP00000308938	PLG	32	0.491	929	23
ENSP00000306245	FOS	129	0.507	994	0
ENSP00000228307	PXN	20	0.509	997	25
ENSP00000300574	CRK	13	0.51	993	45
ENSP00000264708	POMC	1	0.516	977	0
ENSP00000302530	BUB1	100	0.524	741	28
ENSP00000326366	PSEN1	19	0.528	999	22

ENSP00000228872	CDKN1B	5	0.529	999	22
ENSP00000384273	RELA	24	0.529	953	25
ENSP00000370473	IGFBP3	30	0.534	990	21
ENSP00000337825	LCK	103	0.539	999	576
ENSP00000209728	CDC6	15	0.546	834	23
ENSP00000219476	TSC2	25	0.547	968	25
ENSP00000315859	RNPS1	1	0.548	161	23
ENSP00000315702	MOB4	6	0.567	532	23
ENSP00000329623	BCL2	21	0.567	999	57
ENSP00000367207	MYC	19	0.569	996	23
ENSP00000264033	CBL	662	0.577	999	37
ENSP00000216225	RBX1	19	0.591	999	25
ENSP00000302665	IGF1	29	0.596	992	23
ENSP00000371067	JAK2	24	0.604	999	160
ENSP00000252102	NDUFA2	6	0.606	547	20
ENSP00000314458	CDC42	154	0.606	993	274
ENSP00000297494	NOS3	12	0.609	945	23
ENSP00000258962	SRSF1	1	0.61	724	22
ENSP00000398597	EXOSC6	6	0.612	0	0
ENSP00000304895	IRS1	77	0.613	999	0

ENSP00000223023	WASL	34	0.623	934	22
ENSP00000314491	SRRT	21	0.635	204	0
ENSP00000354961	MT-ND4	1	0.635	0	0
ENSP00000262320	AXIN1	89	0.638	948	0
ENSP00000354554	MT-CYB	1	0.64	953	0
ENSP00000221494	SF3A2	139	0.657	0	23
ENSP00000363822	AR	54	0.671	997	25
ENSP00000362649	HDAC1	142	0.69	999	0
ENSP00000312735	POLR2B	13	0.695	343	28
ENSP00000287820	PPARG	19	0.697	902	23
ENSP00000263253	EP300	176	0.735	999	77
ENSP00000329357	SP1	7	0.748	999	120
ENSP00000376076	SUMO1	5	0.749	999	20
ENSP00000357656	FYN	68	0.751	993	749
ENSP00000339007	GRB2	239	0.755	995	92
ENSP00000384675	SOS1	81	0.765	956	29
ENSP00000307863	U2AF2	113	0.768	260	22
ENSP00000274335	PIK3R1	61	0.773	999	52
ENSP00000266970	CDK2	88	0.779	999	245
ENSP00000365891	WAS	8	0.781	984	24

ENSP00000292644	PSMC2	146	0.797	899	22
ENSP00000360683	PTPN1	3	0.798	999	26
ENSP00000263309	CLNS1A	21	0.801	232	0
ENSP00000319169	PRMT5	21	0.809	981	25
ENSP00000206249	ESR1	839	0.81	999	0
ENSP00000215829	SNRPD3	23	0.815	266	0
ENSP00000227378	HSPA8	21	0.819	981	26
ENSP00000274255	SKP2	5	0.82	985	27
ENSP00000287936	HMGCR	12	0.827	435	26
ENSP00000162330	BCAR1	11	0.833	999	33
ENSP00000303830	INSR	26	0.837	962	186
ENSP00000252622	LSM7	6	0.839	491	20
ENSP00000348986	INS-IGF2	26	0.847	973	20
ENSP00000324806	GSK3B	11	0.856	995	119
ENSP00000344818	UBC	3270	0.866	999	0
ENSP00000270202	AKT1	223	0.872	999	62
ENSP00000365439	HNRNPK	113	0.877	991	26
ENSP00000248566	SHFM1	13	0.88	215	18
ENSP00000251849	RAF1	268	0.885	975	155
ENSP00000309845	HRAS	495	0.887	999	67

ENSP00000278616	ATM	4	0.897	999	50
ENSP00000354558	MTOR	1	0.905	998	57
ENSP00000361423	ABL1	7	0.917	995	356
ENSP00000313829	KHDRBS1	92	0.926	999	0
ENSP00000269571	ERBB2	24	0.936	999	191
ENSP00000320940	NCOA1	32	0.952	915	25
ENSP00000341189	PTK2	34	0.96	999	209
ENSP00000335153	HSP90AA1	797	0.992	999	27

2. 64 shortest path chemicals

Pubchem ID	Chemical name	Betweenness	Permutation FDR	MIS
CID000001032	Propionic acid	139	<0.001	987
CID000440641	β-D-Fructose 6-phosphate	139	0.001	905
CID000023925	Iron	658	0.002	994
CID000001132	thiamine pyrophosphate	276	0.004	360
CID000002733	chlorzoxazone	1	0.005	322
CID000004055	menadione	117	0.007	547
CID000001135	thymine	1	0.008	835
CID000003559	haloperidol	71	0.009	660
CID000000979	p-hydroxyphenylpyruvate	135	0.013	0

CID000003776	isopropanol	108	0.013	965
CID000000311	citric acid	142	0.018	583
CID000643975	flavin adenine dinucleotide	158	0.022	900
CID000122357	erythrose 4-phosphate	2	0.024	906
CID000439176	5'-methylthioadenosine	78	0.027	196
CID000005035	raloxifene	86	0.028	239
CID000004174	metyrapone	1	0.031	236
CID000001103	spermine	2	0.038	364
CID000000679	Dimethyl sulfoxide	14	0.048	970
CID000005789	thymidine	3	0.049	729
CID000000190	adenine	62	0.061	953
CID000001005	phosphoenolpyruvate	136	0.066	277
CID000145742	L-proline	1	0.067	254
CID000000896	melatonin	5	0.07	925
CID000023994	zinc	804	0.072	996
CID000005961	glutamine	136	0.079	752
CID000439153	reduced nicotinamide adenine dinucleotide	135	0.088	918
CID000023930	manganese	90	0.09	947
CID000033032	glutamic acid	172	0.1	934

CID005280360	PGE2	138	0.103	940
CID000065063	dUMP	4	0.108	977
CID000004763	phenobarbital	2	0.129	393
CID000000177	acetaldehyde	135	0.13	996
CID000444493	acetyl-CoA	131	0.132	958
CID000124886	Glutathione	136	0.138	900
CID000003032	diclofenac	1	0.155	350
CID000001102	spermidine	76	0.168	428
CID000006083	adenosine monophosphate	150	0.178	325
CID000087642	coenzyme A	192	0.198	952
CID000005950	L-alanine	2	0.206	324
CID000000271	calcium	1692	0.216	968
CID000000681	dopamine	44	0.265	741
CID000000222	ammonia	5	0.284	960
CID000000977	oxygen	356	0.29	975
CID000000888	magnesium	367	0.408	950
CID000444899	arachidonic acid	24	0.431	482
CID000006013	testosterone	54	0.437	486
CID000006022	adenosine diphosphate	141	0.447	990
CID002733525	tamoxifen	119	0.473	711

CID000000753	glycerol	347	0.491	975
CID000000051	alpha-ketoglutarate	13	0.507	946
CID000003715	indomethacin	27	0.535	547
CID000000283	formate	87	0.544	983
CID000006322	arginine	12	0.567	944
CID000439155	AdoHcy	25	0.641	914
CID000001003	phosphate	1	0.643	978
CID000005892	beta-NAD	115	0.682	924
CID000005885	NADP	34	0.694	993
CID000000961	hydroxyl radicals	32	0.703	970
CID000000312	chloride	131	0.73	957
CID000000750	glycine	2	0.74	976
CID000000923	sodium	135	0.818	955
CID000005957	adenosine triphosphate	2157	0.843	974
CID000006830	guanosine triphosphate	433	0.883	929
CID000008977	guanosine diphosphate	4	0.987	901

Supplementary Material IV. KEGG pathways and GO terms analysis of shortest path genes

Category	Term	Count	%	PValue	Genes	List Total	Pop Hits	Pop Total	Fold Enrichment	Bonferroni	Benjamini	FDR
GOTERM_BP_FAT	GO:0002474~antigen processing and presentation of peptide antigen via MHC class I	5	4.16667	8.44E-06	HFE, HLA-C, CALR, TAPBP, B2M	109	17	13528	36.502968	0.01256	0.012563481	0.014
GOTERM_BP_FAT	GO:0032269~negative regulation of cellular protein metabolic process	10	8.33333	1.35E-05	PRKCA, ANAPC1, PSMB5, A2M, PSMA4, BUB1B, IGF2BP2, CALR, BUB3	109	180	13528	6.8950051	0.02004	0.01006908	0.0224
GOTERM_BP_FAT	GO:0051248~negative regulation of protein metabolic process	10	8.33333	1.83E-05	PRKCA, ANAPC1, PSMB5, A2M, PSMA4, BUB1B, IGF2BP2, CALR, BUB3,	109	187	13528	6.6369033	0.02708	0.009109843	0.0304
GOTERM_BP_FAT	GO:0060284~regulation of cell development	10	8.33333	3.79E-05	RTN4, TIAM1, DRD2, GRIN1, EDN1, RTN4R, MBNL3, NGFR, CDH2, CALR	109	205	13528	6.0541508	0.05515	0.014081353	0.0629

GOTERM_BP_FAT	GO:0010033~response to organic substance	18	15	5.40E-05	PRKCA, A2M, UQCRC1, DRD2, GRIN1, PRKCI, GRIN3A, LIN28A, B2M, CCNE1, SLC1A2, TFRC, TICAM1, TFF1, PARP1,	109	721	13528	3.0984489	0.07772	0.016050263	0.0897
GOTERM_BP_FAT	GO:0048002~antigen processing and presentation of peptide antigen	5	4.16667	6.79E-05	HFE, HLA-C, CALR, TAPBP, B2M	109	28	13528	22.162516	0.09666	0.016800152	0.1127
GOTERM_BP_FAT	GO:0050770~regulation of axonogenesis	6	5	8.89E-05	RTN4, TIAM1, GRIN1, RTN4R, NGFR, CDH2,	109	57	13528	13.06422	0.12473	0.018852471	0.1476
GOTERM_BP_FAT	GO:0006955~immune response	17	14.1667	1.11E-04	CXCL9, CCL19, HFE, HLA-C, NFKB2, PSMB8, TAPBP, B2M, LILRB1, TICAM1, BCL3, CD79A, KIR2DL3, KIR3DL1, CD27, BLNK,	109	690	13528	3.0577849	0.15372	0.020647149	0.1849

GOTERM_BP_FAT	GO:0051301~cell division	11	9.16667	1.23E-04	PARD6A, ANAPC1, CCNE1, PPP1CA, ARHGEF2, PARD3, CDK11A, ZWINT, BUB1B, CCNA2, ARHGEF11	109	295	13528	4.6278339	0.16804	0.020233223	0.2038
GOTERM_BP_FAT	GO:0051960~regulation of nervous system development	9	7.5	1.49E-04	PRKCA, RTN4, TIAM1, DRD2, GRIN1, RTN4R, NGFR, CDH2, CALR	109	192	13528	5.8176606	0.20033	0.022107988	0.2476
GOTERM_BP_FAT	GO:0045471~response to ethanol	6	5	1.55E-04	PRKCA, CCNE1, DRD2, GRIN1, GRIN3A, CD27	109	64	13528	11.635321	0.20714	0.020879345	0.2571
GOTERM_BP_FAT	GO:0043065~positive regulation of apoptosis	13	10.8333	1.61E-04	PRKCA, ARHGEF2, PDIA3, MMP9, GRIN1, ECT2, ARHGEF11, TIAM1, TICAM1, CD2, BCL3, NGFR, CD27	109	430	13528	3.7521656	0.21445	0.019913243	0.2673

GOTERM_BP_FAT	GO:0051436~negative regulation of ubiquitin-protein ligase activity during mitotic cell cycle	6	5	1.67E-04	ANAPC1, PSMB5, PSMA4, BUB1B, BUB3, PSMB8	109	65	13528	11.456316	0.22109	0.019036773	0.2767
GOTERM_BP_FAT	GO:0031145~anaphase-promoting complex-dependent proteasomal ubiquitin-dependent protein catabolic process	6	5	1.67E-04	ANAPC1, PSMB5, PSMA4, BUB1B, BUB3, PSMB8	109	65	13528	11.456316	0.22109	0.019036773	0.2767
GOTERM_BP_FAT	GO:0043068~positive regulation of programmed cell death	13	10.8333	1.72E-04	PRKCA, ARHGEF2, PDIA3, MMP9, GRIN1, ECT2, ARHGEF11, TIAM1, TICAM1, CD2, BCL3, NGFR, CD27	109	433	13528	3.726169	0.22701	0.018223554	0.2851
GOTERM_BP_FAT	GO:0010942~positive regulation of cell death	13	10.8333	1.79E-04	PRKCA, ARHGEF2, PDIA3, MMP9, GRIN1, ECT2, ARHGEF11, TIAM1, TICAM1, CD2, BCL3, NGFR, CD27	109	435	13528	3.7090372	0.23565	0.017755795	0.2976

GOTERM_BP_FAT	GO:0051352~negative regulation of ligase activity	6	5	1.93E-04	ANAPC1, PSMB5, PSMA4, BUB1B, BUB3, PSMB8	109	67	13528	11.114337	0.2506	0.017868554	0.3194
GOTERM_BP_FAT	GO:0051444~negative regulation of ubiquitin-protein ligase activity	6	5	1.93E-04	ANAPC1, PSMB5, PSMA4, BUB1B, BUB3, PSMB8	109	67	13528	11.114337	0.2506	0.017868554	0.3194
GOTERM_BP_FAT	GO:0010975~regulation of neuron projection development	6	5	2.37E-04	RTN4, TIAM1, GRIN1, RTN4R, NGFR, CDH2	109	70	13528	10.638008	0.29865	0.020651391	0.3926
GOTERM_CC_FAT	GO:0043235~receptor complex	7	5.83333	3.08E-04	ITGAX, ITGA8, GRIN1, ITGB2, CD79A, GRIN3A, ITGAM	102	116	12782	7.5620352	0.06764	0.067642778	0.394
GOTERM_CC_FAT	GO:0031981~nuclear lumen	25	20.8333	3.10E-04	POLR2H, EZH2, CTCF, NFKB2, LIN28A, RPA3, ZNF207, CCNE1, SAFB, ACTL6A, MBNL3, CCNA2, BUB3, ANAPC1, EPAS1, CCNL1, MCM2, MCM6, ARHGEF11, SUZ12,	102	1450	12782	2.1605815	0.06791	0.034549741	0.3955

GOTERM_BP_FAT	GO:0051439~regulation of ubiquitin-protein ligase activity during mitotic cell cycle	6	5	2.53E-04	ANAPC1, PSMB5, PSMA4, BUB1B, BUB3, PSMB8	109	71	13528	10.488177	0.31561	0.0208477	0.4197
GOTERM_BP_FAT	GO:0008624~induction of apoptosis by extracellular signals	7	5.83333	2.71E-04	PRKCA, ARHGEF2, TIAM1, TICAM1, NGFR, ECT2, ARHGEF11	109	112	13528	7.7568807	0.33416	0.02117784	0.45
GOTERM_MF_FAT	GO:0008599~protein phosphatase type 1 regulator activity	3	2.5	3.56E-04	PPP1CA, PPP1R7, PPP1R8	102	4	12983	95.463235	0.11714	0.117139155	0.4857
GOTERM_CC_FAT	GO:0009897~external side of plasma membrane	8	6.66667	3.92E-04	PARD3, LRP1, ITGAX, CD2, CD79A, CALR, KLRD1, ITGAM	102	170	12782	5.8971165	0.08513	0.029221435	0.5002
GOTERM_BP_FAT	GO:0031397~negative regulation of protein ubiquitination	6	5	3.07E-04	ANAPC1, PSMB5, PSMA4, BUB1B, BUB3, PSMB8	109	74	13528	10.06298	0.36897	0.022757273	0.5093
GOTERM_BP_FAT	GO:0050767~regulation of neurogenesis	8	6.66667	3.64E-04	RTN4, TIAM1, DRD2, GRIN1, RTN4R, NGFR, CDH2, CALR	109	166	13528	5.9812092	0.42054	0.025649013	0.6033
GOTERM_BP_FAT	GO:0010769~regulation of cell morphogenesis involved in differentiation	6	5	3.70E-04	RTN4, TIAM1, GRIN1, RTN4R, NGFR, CDH2	109	77	13528	9.6709162	0.42543	0.024873013	0.6126

GOTERM_BP_FAT	GO:0031400~negative regulation of protein modification process	7	5.83333	3.76E-04	PRKCA, ANAPC1, PSMB5, PSMA4, BUB1B, BUB3, PSMB8	109	119	13528	7.3005936	0.43106	0.024222671	0.6235
GOTERM_CC_FAT	GO:0005654~nucleoplasm	18	15	5.10E-04	ANAPC1, POLR2H, EPAS1, EZH2, CCNL1, CTCF, MCM2, NFKB2, MCM6, RPA3, SUZ12, CCNE1, PPP1CA, PPP1R8, ACTL6A,	102	882	12782	2.557423	0.10928	0.028516955	0.6501
GOTERM_BP_FAT	GO:0051438~regulation of ubiquitin-protein ligase activity	6	5	3.93E-04	ANAPC1, PSMB5, PSMA4, BUB1B, BUB3, PSMB8	109	78	13528	9.5469301	0.44477	0.02421712	0.6504
GOTERM_BP_FAT	GO:0032268~regulation of cellular protein metabolic process	13	10.8333	3.93E-04	ANAPC1, PRKCA, A2M, EDN1, IGF2BP2, ITGB2, CALR, PSMB8, PSMB5, PSMA4, BCL3	109	474	13528	3.4038633	0.44503	0.023278482	0.6509

GOTERM_BP_FAT	GO:0007049~cell cycle	17	14.1667	4.21E-04	PARD6A, ANAPC1, ARHGEF2, PARD3, MCM2, CALR, PSMB8, MCM6, PSMB5, CCNE1, PPP1CA, CDK11A, PSMA4, ZWINT,	109	776	13528	2.7189066	0.46809	0.023987374	0.6976
GOTERM_CC_FAT	GO:0005829~cytosol	23	19.1667	5.89E-04	PRKCA, ANAPC1, ARHGEF2, TALDO1, PFKFB4, PRKCI, NFKB2, CALR, ECT2, PSMB8, ARHGEF11, PSMB5, TYMS, CCNE1, PPP1CA, TYMP, TIAM1, RRM2, PSMA4, TICAM1, BUB1B, NGFR, BUB3	102	1330	12782	2.1670795	0.12527	0.026412226	0.7515

GOTERM_BP_FAT	GO:0030005~cellular di-, tri-valent inorganic cation homeostasis	9	7.5	4.63E-04	PRKCA, TFRC, DRD2, GRIN1, EDN1, HFE, CCL19, CXCR3, CALR	109	227	13528	4.9206644	0.5006	0.02538843	0.7671
GOTERM_BP_FAT	GO:0051340~regulation of ligase activity	6	5	4.68E-04	ANAPC1, PSMB5, PSMA4, BUB1B, BUB3, PSMB8	109	81	13528	9.1933401	0.50377	0.024715031	0.7741
GOTERM_CC_FAT	GO:0044459~plasma membrane part	32	26.6667	6.16E-04	PARD3, DRD2, HFE, ITGB2, GRIN3A, CXCR3, CDH2, CALR, ITGAM, B2M, SLC1A2, ITGAX, CD2, KLRD1, CD27, PARD6A, ARHGEF2, GRIN1, PRKCI, HLA-C, SLC9A3R1, SIGMAR1, CTNNA1, LRP1, TFRC, ITGA8, MTNR1B, NGFR, CD79A, KIR2DL3, KIR3DL1, KLRC1	102	2203	12782	1.8202629	0.13054	0.02304366	0.7853

GOTERM_BP_FAT	GO:0001775~cell activation	10	8.33333	4.80E-04	TICAM1, EDN1, CD2, IRF1, BCL3, NFKB2, CD79A, KIR3DL1, ITGAM, BLNK	109	287	13528	4.3243934	0.51255	0.024473764	0.7937
GOTERM_BP_FAT	GO:0019882~antigen processing and presentation	6	5	5.23E-04	HFE, HLA-C, CALR, PSMB8, TAPBP, B2M	109	83	13528	8.9718139	0.54348	0.02579888	0.8658
GOTERM_CC_FAT	GO:0043233~organelle lumen	28	23.3333	6.97E-04	POLR2H, A2M, PDIA3, EZH2, CTCF, NFKB2, CALR, LIN28A, RPA3, ZNF207, CCNE1, SAFB, ACTL6A, MBNL3, CCNA2, BUB3, ANAPC1, EPAS1, CCNL1, MCM2, ARHGEF11, MCM6, SUZ12, PPP1CA, ATF3, PPP1R8,	102	1820	12782	1.9279035	0.14638	0.022355734	0.888

GOTERM_BP_FAT	GO:0042981~regulation of apoptosis	17	14.1667	6.21E-04	PRKCA, RTN4, ARHGEF2, PDIA3, MMP9, GRIN1, PRKCI, CALR, ECT2, ARHGEF11, TIAM1, TICAM1, CD2, BCL3, NGFR, CD27, GSTP1	109	804	13528	2.6242184	0.60537	0.029548328	1.0258
GOTERM_BP_FAT	GO:0022604~regulation of cell morphogenesis	7	5.83333	6.28E-04	RTN4, TIAM1, GRIN1, RTN4R, ITGB2, NGFR, CDH2	109	131	13528	6.631837	0.60957	0.028963099	1.0376
GOTERM_BP_FAT	GO:0055066~di-, tri-valent inorganic cation homeostasis	9	7.5	6.51E-04	PRKCA, TFRC, DRD2, GRIN1, EDN1, HFE, CCL19, CXCR3, CALR	109	239	13528	4.6736018	0.62297	0.029125616	1.0759
GOTERM_BP_FAT	GO:0045664~regulation of neuron differentiation	7	5.83333	6.80E-04	RTN4, TIAM1, GRIN1, RTN4R, NGFR, CDH2, CALR	109	133	13528	6.5321101	0.63894	0.029517589	1.1234

GOTERM_BP_FAT	GO:0043067~regulation of programmed cell death	17	14.1667	6.91E-04	PRKCA, RTN4, ARHGEF2, PDIA3, MMP9, GRIN1, PRKCI, CALR, ECT2, ARHGEF11, TIAM1, TICAM1, CD2, BCL3, NGFR, CD27, GSTP1	109	812	13528	2.598364	0.64468	0.029131092	1.141
GOTERM_BP_FAT	GO:0045321~leukocyte activation	9	7.5	7.06E-04	TICAM1, EDN1, CD2, IRF1, BCL3, CD79A, KIR3DL1, ITGAM, BLNK	109	242	13528	4.6156646	0.65299	0.028972302	1.1669
GOTERM_BP_FAT	GO:0010941~regulation of cell death	17	14.1667	7.18E-04	PRKCA, RTN4, ARHGEF2, PDIA3, MMP9, GRIN1, PRKCI, CALR, ECT2, ARHGEF11, TIAM1, TICAM1, CD2, BCL3, NGFR, CD27, GSTP1	109	815	13528	2.5887995	0.65926	0.028678672	1.1869
GOTERM_BP_FAT	GO:0031344~regulation of cell projection organization	6	5	7.20E-04	RTN4, TIAM1, GRIN1, RTN4R, NGFR, CDH2	109	89	13528	8.3669725	0.66024	0.028008458	1.19

GOTERM_CC_FAT	GO:0031974~membrane-enclosed lumen	28	23.3333	9.45E-04	POLR2H, A2M, PDIA3, EZH2, CTCF, NFKB2, CALR, LIN28A, RPA3, ZNF207, CCNE1, SAFB, ACTL6A, MBNL3, CCNA2, BUB3, ANAPC1, EPAS1, CCNL1, MCM2, ARHGEF11, MCM6, SUZ12, PPP1CA, ATF3, PPP1R8,	102	1856	12782	1.8905088	0.1932	0.026477907	1.2026
GOTERM_BP_FAT	GO:0000278~mitotic cell cycle	11	9.16667	7.43E-04	ANAPC1, PSMB5, CCNE1, ARHGEF2, CDK11A, PSMA4, ZWINT, BUB1B	109	370	13528	3.6897595	0.67181	0.028164088	1.228

GOTERM_CC_FAT	GO:0070013~intracellular organelle lumen	27	22.5	0.001129	POLR2H, PDIA3, EZH2, CTCF, NFKB2, CALR, LIN28A, RPA3, ZNF207, CCNE1, SAFB, ACTL6A, MBNL3, CCNA2, BUB3, ANAPC1, EPAS1, CCNL1, MCM2, ARHGEF11, MCM6, SUZ12, PPP1CA, ATF3,	102	1779	12782	1.9018947	0.22613	0.028081726	1.4344
GOTERM_CC_FAT	GO:0031226~intrinsic to plasma membrane	21	17.5	0.001164	PARD3, DRD2, GRIN1, HFE, ITGB2, HLA-C, GRIN3A, CXCR3, SIGMAR1, ITGAM, LRP1, ITGAX, TFRC, ITGA8, MTNR1B, CD2, NGFR, KIR2DL3, KIR3DL1, CD27, KLRC1	102	1215	12782	2.1659162	0.23228	0.026086197	1.4787

GOTERM_BP_FAT	GO:0030003~cellular cation homeostasis	9	7.5	9.67E-04	PRKCA, TFRC, DRD2, GRIN1, EDN1, HFE, CCL19, CXCR3, CALR	109	254	13528	4.3976017	0.76527	0.035583962	1.5944
GOTERM_BP_FAT	GO:0006917~induction of apoptosis	10	8.33333	0.001041	PRKCA, ARHGEF2, TIAM1, TICAM1, CD2, BCL3, NGFR, ECT2, CD27, ARHGEF11	109	320	13528	3.8784404	0.79003	0.037352624	1.716
GOTERM_BP_FAT	GO:0012502~induction of programmed cell death	10	8.33333	0.001064	PRKCA, ARHGEF2, TIAM1, TICAM1, CD2, BCL3, NGFR, ECT2, CD27, ARHGEF11	109	321	13528	3.866358	0.79715	0.037270982	1.7536
GOTERM_CC_FAT	GO:0008305~integrin complex	4	3.33333	0.001507	ITGAX, ITGA8, ITGB2, ITGAM	102	29	12782	17.284652	0.28993	0.030646778	1.9112
GOTERM_BP_FAT	GO:0031396~regulation of protein ubiquitination	6	5	0.001219	ANAPC1, PSMB5, PSMA4, BUB1B, BUB3, PSMB8	109	100	13528	7.4466055	0.83919	0.041610793	2.0063
GOTERM_BP_FAT	GO:0048585~negative regulation of response to stimulus	6	5	0.001219	PRKCA, AMBP, A2M, DRD2, GRIN1, GRIN3A	109	100	13528	7.4466055	0.83919	0.041610793	2.0063

GOTERM_BP_FAT	GO:0010498~proteasomal protein catabolic process	6	5	0.001332	ANAPC1, PSMB5, PSMA4, BUB1B, BUB3, PSMB8	109	102	13528	7.3005936	0.86422	0.044365395	2.19
GOTERM_BP_FAT	GO:0043161~proteasomal ubiquitin-dependent protein catabolic process	6	5	0.001332	ANAPC1, PSMB5, PSMA4, BUB1B, BUB3, PSMB8	109	102	13528	7.3005936	0.86422	0.044365395	2.19
GOTERM_MF_FAT	GO:0008121~ubiquinol-cytochrome-c reductase activity	3	2.5	0.001627	UQCRC1, UQCRH, UQCRQ	102	8	12983	47.731618	0.43451	0.248008699	2.203
GOTERM_MF_FAT	GO:0016681~oxidoreductase activity, acting on diphenols and related substances as donors, cytochrome as	3	2.5	0.001627	UQCRC1, UQCRH, UQCRQ	102	8	12983	47.731618	0.43451	0.248008699	2.203
GOTERM_BP_FAT	GO:0007626~locomotory behavior	9	7.5	0.001569	PRKCA, TYMP, DRD2, GRIN1, CXCL9, CCL19, ITGB2, CXCR3	109	274	13528	4.0766089	0.90478	0.050915504	2.5742
GOTERM_CC_FAT	GO:0042824~MHC class I peptide loading complex	3	2.5	0.002147	CALR, PSMB8, TAPBP	102	9	12782	41.771242	0.38602	0.03983472	2.7117

GOTERM_CC_FAT	GO:0005887~integral to plasma membrane	20	16.6667	0.002196	DRD2, GRIN1, HFE, ITGB2, HLA-C, GRIN3A, CXCR3, SIGMAR1, ITGAM, LRP1, ITGAX, TFRC, ITGA8, MTNR1B, CD2, NGFR, KIR2DL3, KIR3DL1,	102	1188	12782	2.1096587	0.39285	0.037655656	2.773
GOTERM_MF_FAT	GO:0016679~oxidoreductase activity, acting on diphenols and related substances as donors	3	2.5	0.002082	UQCRC1, UQCRH, UQCRQ	102	9	12983	42.428105	0.5178	0.215829941	2.81
GOTERM_BP_FAT	GO:0042330~taxis	7	5.83333	0.001767	PRKCA, TYMP, CXCL9, CCL19, ITGB2, CXCR3, ITGAM,	109	160	13528	5.4298165	0.92934	0.055979125	2.896
GOTERM_BP_FAT	GO:0006935~chemotaxis	7	5.83333	0.001767	PRKCA, TYMP, CXCL9, CCL19, ITGB2, CXCR3, ITGAM,	109	160	13528	5.4298165	0.92934	0.055979125	2.896

GOTERM_BP_FAT	GO:0007242~intracellular signaling cascade	21	17.5	0.002051	PRKCA, ARHGEF2, DRD2, EDN1, PRKCI, CALR, ECT2, TANK, ARHGEF11, TYMS, CCNE1, TIAM1, ZWINT, TICAM1, MTNR1B, BUB1B, BCL3, CCNA2, CD27, BLNK, ARHGDIB	109	1256	13528	2.0750891	0.95384	0.063344238	3.3534
GOTERM_BP_FAT	GO:0055080~cation homeostasis	9	7.5	0.002052	PRKCA, TFRC, DRD2, GRIN1, EDN1, HFE, CCL19, CXCR3, CALR	109	286	13528	3.9055623	0.95393	0.062105334	3.3555
GOTERM_BP_FAT	GO:0010605~negative regulation of macromolecule metabolic process	15	12.5	0.002069	PRKCA, ANAPC1, A2M, EDN1, IGF2BP2, CTCF, LIN28A, CALR, PSMB8, SUZ12, PSMB5, PSMA4	109	734	13528	2.5363098	0.95509	0.06136419	3.3827

GOTERM_BP_FAT	GO:0000902~cell morphogenesis	10	8.33333	0.00217	PRKCA, ARHGEF2, PARD3, NDN, DRD2, RTN4R, PRKCI, NGFR, ECT2, ARHGEF11	109	356	13528	3.4862385	0.9614	0.063014628	3.5447
GOTERM_BP_FAT	GO:0043414~biopolymer methylation	5	4.16667	0.00223	ATRX, SUZ12, EZH2, CTCF, SETD2	109	69	13528	8.9934849	0.9647	0.06346546	3.6406
GOTERM_BP_FAT	GO:0031399~regulation of protein modification process	9	7.5	0.002487	PRKCA, ANAPC1, PSMB5, PSMA4, EDN1, BUB1B, ITGB2, BUB3, PSMB8	109	295	13528	3.7864096	0.97601	0.069224149	4.0525
GOTERM_BP_FAT	GO:0042592~homeostatic process	15	12.5	0.002554	PRKCA, PARD3, PDIA3, EPAS1, DRD2, GRIN1, EDN1, CCL19, HFE, CXCR3, CALR, SLC9A3R1, TFRC, TFF1, PARP1	109	751	13528	2.4788966	0.97831	0.069728258	4.1593
GOTERM_BP_FAT	GO:0060134~prepulse inhibition	3	2.5	0.002725	DRD2, GRIN1, GRIN3A	109	10	13528	37.233028	0.98323	0.072911968	4.4325

GOTERM_BP_FAT	GO:0006873~cellular ion homeostasis	10	8.33333	0.00302	PRKCA, TFRC, DRD2, GRIN1, EDN1, HFE, CCL19, CXCR3, SLC9A3R1, CALR	109	374	13528	3.3184517	0.98923	0.079084545	4.901
GOTERM_BP_FAT	GO:0032259~methylation	5	4.16667	0.003171	ATRX, SUZ12, EZH2, CTCF, SETD2	109	76	13528	8.1651376	0.99142	0.081456769	5.1399
GOTERM_BP_FAT	GO:0010604~positive regulation of macromolecule metabolic process	16	13.3333	0.003269	PRKCA, ANAPC1, EPAS1, GRIN1, CTCF, CALR, PSMB8, PRPF6, ARHGEF11, PSMB5, CCNE1, PSMA4, TICAM1, IRF1,	109	857	13528	2.3171079	0.99259	0.082458661	5.2946
GOTERM_BP_FAT	GO:0055082~cellular chemical homeostasis	10	8.33333	0.003356	PRKCA, TFRC, DRD2, GRIN1, EDN1, HFE, CCL19, CXCR3, SLC9A3R1, CALR	109	380	13528	3.266055	0.9935	0.083159477	5.4314
GOTERM_BP_FAT	GO:0006874~cellular calcium ion homeostasis	7	5.83333	0.003457	PRKCA, DRD2, GRIN1, EDN1, CCL19, CXCR3, CALR	109	183	13528	4.7473806	0.99442	0.084172817	5.5908

GOTERM_BP_FAT	GO:0055074~calcium ion homeostasis	7	5.83333	0.003945	PRKCA, DRD2, GRIN1, EDN1, CCL19, CXCR3, CALR	109	188	13528	4.6211204	0.99732	0.093978326	6.3559
GOTERM_BP_FAT	GO:0019725~cellular homeostasis	11	9.16667	0.004063	PRKCA, PDIA3, TFRC, DRD2, GRIN1, EDN1, HFE, CCL19, CXCR3, SLC9A3R1, CALR	109	466	13528	2.9296374	0.99776	0.095148736	6.5401
GOTERM_BP_FAT	GO:0007610~behavior	11	9.16667	0.00425	PRKCA, TYMP, SLC1A2, DRD2, ITGA8, GRIN1, CXCL9, CCL19, ITGB2	109	469	13528	2.9108977	0.99831	0.097792576	6.8313
GOTERM_BP_FAT	GO:0032989~cellular component morphogenesis	10	8.33333	0.004469	PRKCA, ARHGEF2, PARD3, NDN, DRD2, RTN4R, PRKCI, NGFR, ECT2, ARHGEF11	109	397	13528	3.1261988	0.99878	0.101021775	7.1705
GOTERM_BP_FAT	GO:0006875~cellular metal ion homeostasis	7	5.83333	0.00483	PRKCA, DRD2, GRIN1, EDN1, CCL19, CXCR3, CALR	109	196	13528	4.4325033	0.99929	0.107139089	7.7283
GOTERM_BP_FAT	GO:0007093~mitotic cell cycle checkpoint	4	3.33333	0.004843	ZWINT, BUB1B, CCNA2, BUB3	109	43	13528	11.545125	0.99931	0.105845756	7.748

GOTERM_BP_FAT	GO:0009719~response to endogenous stimulus	10	8.33333	0.005083	PRKCA, CCNE1, A2M, SLC1A2, DRD2, GRIN1, PRKCI, TFF1, PARP1	109	405	13528	3.0644467	0.99952	0.10923468	8.1181
GOTERM_BP_FAT	GO:0046649~lymphocyte activation	7	5.83333	0.005197	CD2, IRF1, BCL3, CD79A, KIR3DL1, ITGAM, BLNK	109	199	13528	4.3656816	0.99959	0.109963486	8.2917
GOTERM_BP_FAT	GO:0048015~phosphoinositide-mediated signaling	5	4.16667	0.005359	TYMS, DRD2, ZWINT, EDN1, BUB1B	109	88	13528	7.0517098	0.99968	0.111638101	8.5401
GOTERM_BP_FAT	GO:0022402~cell cycle process	12	10	0.005411	ANAPC1, PSMB5, CCNE1, ARHGEF2, CDK11A, PSMA4, ZWINT, BUB1B, CALR, CCNA2, BUB3	109	565	13528	2.6359666	0.9997	0.111122014	8.6194
GOTERM_BP_FAT	GO:0050801~ion homeostasis	10	8.33333	0.005415	PRKCA, TFRC, DRD2, GRIN1, EDN1, HFE, CCL19, CXCR3, SLC9A3R1, CALR	109	409	13528	3.0344766	0.99971	0.10969091	8.6247
GOTERM_BP_FAT	GO:0055065~metal ion homeostasis	7	5.83333	0.005991	PRKCA, DRD2, GRIN1, EDN1, CCL19, CXCR3, CALR	109	205	13528	4.2379056	0.99988	0.119078394	9.5008

GOTERM_MF_FAT	GO:0019888~protein phosphatase regulator activity	4	3.33333	0.008078	PPP1CA, PPP1R7, PPP1R8, PPP2R5A	102	53	12983	9.6063633	0.94151	0.508212621	10.5
GOTERM_BP_FAT	GO:0043086~negative regulation of catalytic activity	8	6.66667	0.006732	PRKCA, ANAPC1, PSMB5, DRD2, PSMA4, BUB1B, BUB3, PSMB8	109	277	13528	3.584407	0.99996	0.131107044	10.615
GOTERM_CC_FAT	GO:0042995~cell projection	13	10.8333	0.008738	RTN4, PARD6A, PARD3, DRD2, GRIN1, CDH2, GRIN3A, SIGMAR1, SLC9A3R1, SLC1A2, PPP1CA, LRP1, ITGA8	102	697	12782	2.3372718	0.86361	0.132640895	10.621
GOTERM_BP_FAT	GO:0001964~startle response	3	2.5	0.007044	DRD2, GRIN1, GRIN3A	109	16	13528	23.270642	0.99997	0.13503125	11.08
GOTERM_BP_FAT	GO:0045596~negative regulation of cell differentiation	7	5.83333	0.007673	RTN4, EZH2, RTN4R, MBNL3, NGFR, LIN28A, CALR	109	216	13528	4.0220863	0.99999	0.144377652	12.011
GOTERM_MF_FAT	GO:0019208~phosphatase regulator activity	4	3.33333	0.0094	PPP1CA, PPP1R7, PPP1R8, PPP2R5A	102	56	12983	9.0917367	0.96332	0.483728408	12.118

GOTERM_BP_FAT	GO:0048878~chemical homeostasis	11	9.16667	0.00776	PRKCA, EPAS1, TFRC, DRD2, GRIN1, EDN1, HFE, CCL19, CXCR3, SLC9A3R1, CALR	109	512	13528	2.6664278	0.99999	0.144095681	12.139
GOTERM_BP_FAT	GO:0035023~regulation of Rho protein signal transduction	5	4.16667	0.008094	ARHGEF2, TIAM1, CDH2, ECT2, ARHGEF11	109	99	13528	6.2681865	0.99999	0.148015637	12.629
GOTERM_CC_FAT	GO:0043025~cell soma	6	5	0.010632	RTN4, PPP1CA, LRP1, NDN, ITGA8, GRIN3A	102	168	12782	4.4754902	0.91164	0.149352844	12.781
GOTERM_CC_FAT	GO:0005839~proteasome core complex	3	2.5	0.010706	PSMB5, PSMA4, PSMB8	102	20	12782	18.797059	0.91314	0.141625776	12.865
GOTERM_BP_FAT	GO:0000280~nuclear division	7	5.83333	0.008362	ANAPC1, ARHGEF2, CDK11A, ZWINT, BUB1B, CCNA2, BUB3	109	220	13528	3.9489575	1	0.15071259	13.02
GOTERM_BP_FAT	GO:0007067~mitosis	7	5.83333	0.008362	ANAPC1, ARHGEF2, CDK11A, ZWINT, BUB1B, CCNA2, BUB3	109	220	13528	3.9489575	1	0.15071259	13.02
GOTERM_BP_FAT	GO:0009968~negative regulation of signal transduction	7	5.83333	0.008541	PRKCA, AMBP, LRP1, DRD2, EZH2, NGFR, CALR	109	221	13528	3.9310889	1	0.151876043	13.281

GOTERM_MF_FAT	GO:0004298~threonine-type endopeptidase activity	3	2.5	0.010392	PSMB5, PSMA4, PSMB8	102	20	12983	19.092647	0.97417	0.456315886	13.314
GOTERM_MF_FAT	GO:0070003~threonine-type peptidase activity	3	2.5	0.010392	PSMB5, PSMA4, PSMB8	102	20	12983	19.092647	0.97417	0.456315886	13.314

GOTERM_CC_FAT	GO:0005886~plasma membrane	42	35	0.011294	RTN4, PARD3, DRD2, HFE, ITGB2, GRIN3A, CXCR3, CDH2, CALR, ITGAM, B2M, SLC1A2, ITGAX, TIAM1, CD2, ACTL6A, KLRD1, CD27, BLNK, PARD6A, PRKCA, ARHGEF2, GRIN1, RTN4R, PRKCI, HLA-C, SIGMAR1, SLC9A3R1, CTNNA1, ARHGEF11, LILRB1, AMBP, LRP1, TFRC, ITGA8, MTNR1B, CD79A, NGFR, KIR2DL3	102	3777	12782	1.3934807	0.9241	0.140725149	13.525

GOTERM_CC_FAT	GO:0044427~chromosomal part	9	7.5	0.011329	ATRX, SUZ12, PPP2R5A, ZWINT, BUB1B, CTCF, MCM2, BUB3, RPA3	102	386	12782	2.9218226	0.92471	0.133843953	13.564
GOTERM_BP_FAT	GO:0030593~neutrophil chemotaxis	3	2.5	0.008889	PRKCA, ITGB2, ITGAM	109	18	13528	20.685015	1	0.15574304	13.785
GOTERM_BP_FAT	GO:0032101~regulation of response to external stimulus	6	5	0.008897	PRKCA, A2M, DRD2, GRIN1, EDN1, GRIN3A	109	159	13528	4.6833997	1	0.154091309	13.797
GOTERM_BP_FAT	GO:0051050~positive regulation of transport	7	5.83333	0.008907	PRKCA, SLC1A2, DRD2, EDN1, PRKCI, CALR, CD27	109	223	13528	3.8958325	1	0.152493379	13.811
GOTERM_CC_FAT	GO:0034708~methyltransferase complex	3	2.5	0.011773	SUZ12, PPP1CA, EZH2	102	21	12782	17.901961	0.932	0.131935505	14.059
GOTERM_CC_FAT	GO:0035097~histone methyltransferase complex	3	2.5	0.011773	SUZ12, PPP1CA, EZH2	102	21	12782	17.901961	0.932	0.131935505	14.059
GOTERM_BP_FAT	GO:0000087~M phase of mitotic cell cycle	7	5.83333	0.009094	ANAPC1, ARHGEF2, CDK11A, ZWINT, BUB1B, CCNA2, BUB3	109	224	13528	3.8784404	1	0.153706896	14.081
GOTERM_BP_FAT	GO:0032355~response to estradiol stimulus	4	3.33333	0.009137	PRKCA, CCNE1, TFF1, CCNA2	109	54	13528	9.1933401	1	0.152667378	14.143
GOTERM_BP_FAT	GO:0043062~extracellular structure organization	6	5	0.009842	DRD2, MMP9, ITGA8, BCL3, NFKB2, CDH2	109	163	13528	4.5684696	1	0.161700092	15.152

GOTERM_BP_FAT	GO:0048285~organelle fission	7	5.83333	0.010072	ANAPC1, ARHGEF2, CDK11A, ZWINT, BUB1B, CCNA2, BUB3	109	229	13528	3.7937583	1	0.163400891	15.48
GOTERM_BP_FAT	GO:0043933~macromolecular complex subunit organization	13	10.8333	0.010985	POLR2H, PARD3, HFE, CTCF, CDH2, MCM2, CALR, SLC9A3R1, TAPBP, PRPF6, UQCRH, RRM2, WDR77	109	710	13528	2.2724383	1	0.175023549	16.765
GOTERM_BP_FAT	GO:0007568~aging	5	4.16667	0.011616	PRKCA, SLC1A2, LRP1, TFRC, CALR	109	110	13528	5.6413678	1	0.182232516	17.642
GOTERM_CC_FAT	GO:0033257~Bcl3/NF-kappaB2 complex	2	1.66667	0.015742	BCL3, NFKB2	102	2	12782	125.31373	0.97272	0.164804057	18.372
GOTERM_CC_FAT	GO:0035098~ESC/E(Z) complex	2	1.66667	0.015742	SUZ12, EZH2	102	2	12782	125.31373	0.97272	0.164804057	18.372
GOTERM_BP_FAT	GO:0006730~one-carbon metabolic process	5	4.16667	0.012347	ATRX, SUZ12, EZH2, CTCF, SETD2	109	112	13528	5.5406291	1	0.190616611	18.648
GOTERM_CC_FAT	GO:0000775~chromosome, centromeric region	5	4.16667	0.016738	PPP2R5A, ZWINT, BUB1B, CTCF, BUB3	102	124	12782	5.0529728	0.97833	0.16678123	19.422
GOTERM_BP_FAT	GO:0002027~regulation of heart rate	3	2.5	0.013145	EPAS1, DRD2, EDN1	109	22	13528	16.924103	1	0.199653451	19.733
GOTERM_BP_FAT	GO:0016571~histone methylation	3	2.5	0.013145	SUZ12, EZH2, SETD2	109	22	13528	16.924103	1	0.199653451	19.733

GOTERM_BP_FAT	GO:0006928~cell motion	10	8.33333	0.013676	PRKCA, NDN, DRD2, ITGB2, NGFR, CXCR3, CDH2, ITGAM, ARHGEF11, ARHGDIB	109	475	13528	2.612844	1	0.204828892	20.448
GOTERM_BP_FAT	GO:0051173~positive regulation of nitrogen compound metabolic process	12	10	0.013709	CCNE1, EPAS1, TICAM1, GRIN1, EDN1, IRF1, BCL3, CTCF, CALR, CCNA2, ARHGEF11	109	644	13528	2.3126104	1	0.203268039	20.493
GOTERM_BP_FAT	GO:0007249~I-kappaB kinase/NF-kappaB cascade	4	3.33333	0.013893	TICAM1, BCL3, CD27, TANK	109	63	13528	7.8800058	1	0.203718495	20.739
GOTERM_BP_FAT	GO:0010243~response to organic nitrogen	4	3.33333	0.013893	CCNE1, SLC1A2, DRD2, GRIN1	109	63	13528	7.8800058	1	0.203718495	20.739
GOTERM_MF_FAT	GO:0050681~androgen receptor binding	3	2.5	0.017247	CCNE1, CALR, PRPF6	102	26	12983	14.686652	0.99773	0.580999008	21.175
GOTERM_CC_FAT	GO:0043005~neuron projection	8	6.66667	0.01861	PPP1CA, SLC1A2, LRP1, DRD2, ITGA8, GRIN1, GRIN3A, SIGMAR1	102	342	12782	2.9313152	0.98594	0.176203916	21.363
GOTERM_BP_FAT	GO:0010648~negative regulation of cell communication	7	5.83333	0.014479	PRKCA, AMBP, LRP1, DRD2, EZH2, NGFR, CALR	109	248	13528	3.5031074	1	0.209364784	21.517

GOTERM_BP_FAT	GO:0051130~positive regulation of cellular component organization	6	5	0.01495	PRKCA, TIAM1, EDN1, PRKCI, NGFR, CALR	109	181	13528	4.1141467	1	0.213410106	22.139
GOTERM_MF_FAT	GO:0008134~transcription factor binding	10	8.33333	0.01834	PARD6A, CCNE1, ATF3, EPAS1, WDR77, BCL3, CTCF, NFKB2, PARP1, PRPF6	102	513	12983	2.4811757	0.99846	0.555056408	22.365
GOTERM_BP_FAT	GO:0006954~inflammatory response	8	6.66667	0.015303	A2M, TFRC, AOX1, TICAM1, CXCL9, CCL19, ITGB2	109	325	13528	3.0550176	1	0.21585551	22.6
GOTERM_BP_FAT	GO:0051276~chromosome organization	10	8.33333	0.015477	SUZ12, SAFB, ZWINT, EZH2, ACTL6A, CTCF, MCM2, SETD2, PARP1, BUB3	109	485	13528	2.558971	1	0.216038256	22.827
GOTERM_CC_FAT	GO:0009986~cell surface	8	6.66667	0.020259	PARD3, LRP1, ITGAX, CD2, CD79A, CALR, KLRD1, ITGAM	102	348	12782	2.8807753	0.9904	0.182902583	23.037
GOTERM_CC_FAT	GO:0042612~MHC class I protein complex	3	2.5	0.020447	HFE, HLA-C, B2M	102	28	12782	13.426471	0.99081	0.177490879	23.225
GOTERM_BP_FAT	GO:0002268~follicular dendritic cell differentiation	2	1.66667	0.015904	BCL3, NFKB2	109	2	13528	124.11009	1	0.219312313	23.381

GOTERM_BP_FAT	GO:0002266~follicular dendritic cell activation	2	1.66667	0.015904	BCL3, NFKB2	109	2	13528	124.11009	1	0.219312313	23.381
GOTERM_MF_FAT	GO:0005089~Rho guanyl-nucleotide exchange factor activity	4	3.33333	0.019889	ARHGEF2, TIAM1, ECT2, ARHGEF11	102	74	12983	6.8802332	0.99912	0.542163664	24.024
GOTERM_BP_FAT	GO:0031175~neuron projection development	7	5.83333	0.016684	PRKCA, PARD3, NDN, DRD2, RTN4R, NGFR, GRIN3A	109	256	13528	3.3936353	1	0.226769255	24.384
GOTERM_BP_FAT	GO:0051726~regulation of cell cycle	8	6.66667	0.016756	ZWINT, EDN1, BUB1B, CTCF, NGFR, CALR, CCNA2, BUB3	109	331	13528	2.9996397	1	0.225615656	24.476
GOTERM_BP_FAT	GO:0050771~negative regulation of axonogenesis	3	2.5	0.016808	RTN4, RTN4R, NGFR	109	25	13528	14.893211	1	0.224251318	24.543
GOTERM_MF_FAT	GO:0019899~enzyme binding	10	8.33333	0.020508	PARD6A, LILRB1, A2M, ARHGEF2, LRP1, EPAS1, ITGB2, GRIN3A, CDH2, ECT2	102	523	12983	2.4337345	0.99929	0.51579315	24.678

GOTERM_BP_FAT	GO:0065003~macromolecular complex assembly	12	10	0.017033	POLR2H, PARD3, UQCRH, RRM2, WDR77, HFE, MCM2, CDH2, SLC9A3R1, CALR, PRPF6, TAPBP	109	665	13528	2.2395806	1	0.224936728	24.829
GOTERM_BP_FAT	GO:0051437~positive regulation of ubiquitin-protein ligase activity during mitotic cell cycle	4	3.33333	0.01704	ANAPC1, PSMB5, PSMA4, PSMB8	109	68	13528	7.3005936	1	0.223070766	24.837
GOTERM_BP_FAT	GO:0044092~negative regulation of molecular function	8	6.66667	0.017518	PRKCA, ANAPC1, PSMB5, DRD2, PSMA4, BUB1B, BUB3, PSMB8	109	334	13528	2.9726968	1	0.226660894	25.443
GOTERM_BP_FAT	GO:0008284~positive regulation of cell proliferation	9	7.5	0.017736	PRKCA, SUZ12, ATF3, DRD2, TICAM1, EDN1, NGFR, CALR, CCNA2	109	414	13528	2.6980455	1	0.22721373	25.716
GOTERM_BP_FAT	GO:0048534~hemopoietic or lymphoid organ development	7	5.83333	0.017869	EPAS1, MMP9, IRF1, BCL3, NFKB2, CD79A, BLNK	109	260	13528	3.3414255	1	0.226819582	25.884
GOTERM_BP_FAT	GO:0006260~DNA replication	6	5	0.01807	TYMS, TYMP, RRM2, MCM2, MCM6, RPA3	109	190	13528	3.9192661	1	0.227170885	26.135

GOTERM_CC_FAT	GO:0031252~cell leading edge	5	4.16667	0.023751	PARD6A, SLC1A2, ITGA8, CDH2, SLC9A3R1	102	138	12782	4.5403524	0.99573	0.196083564	26.473
GOTERM_BP_FAT	GO:0007229~integrin-mediated signaling pathway	4	3.33333	0.018401	ITGAX, ITGA8, ITGB2, ITGAM	109	70	13528	7.0920052	1	0.228955806	26.548
GOTERM_BP_FAT	GO:0051443~positive regulation of ubiquitin-protein ligase activity	4	3.33333	0.018401	ANAPC1, PSMB5, PSMA4, PSMB8	109	70	13528	7.0920052	1	0.228955806	26.548
GOTERM_BP_FAT	GO:0048666~neuron development	8	6.66667	0.018843	PRKCA, PARD3, NDN, DRD2, RTN4R, PRKCI, NGFR, GRIN3A	109	339	13528	2.9288517	1	0.231912085	27.095
GOTERM_BP_FAT	GO:0007409~axonogenesis	6	5	0.019198	PRKCA, PARD3, NDN, DRD2, RTN4R, NGFR	109	193	13528	3.8583448	1	0.233875098	27.533

GOTERM_BP_FAT	GO:0007166~cell surface receptor linked signal transduction	24	20	0.019694	PARD3, NDN, DRD2, EDN1, GRIN1, CXCL9, CCL19, ITGB2, GRIN3A, CXCR3, SLC9A3R1, ITGAM, ARHGEF11, LRP1, ITGAX, TIAM1, ITGA8, TICAM1, MTNR1B, CD2, CD79A, KLRD1,	109	1856	13528	1.6048719	1	0.237283491	28.139
GOTERM_BP_FAT	GO:0051351~positive regulation of ligase activity	4	3.33333	0.020553	ANAPC1, PSMB5, PSMA4, PSMB8	109	73	13528	6.800553	1	0.244410867	29.177
GOTERM_BP_FAT	GO:0031328~positive regulation of cellular biosynthetic process	12	10	0.020732	CCNE1, EPAS1, TICAM1, GRIN1, EDN1, IRF1, BCL3, CTCF, CALR, CCNA2, ARHGEF11	109	685	13528	2.1741914	1	0.24436723	29.392
GOTERM_BP_FAT	GO:0002521~leukocyte differentiation	5	4.16667	0.020778	MMP9, IRF1, BCL3, CD79A, BLNK	109	131	13528	4.7370264	1	0.242968927	29.448

GOTERM_BP_FAT	GO:0031345~negative regulation of cell projection organization	3	2.5	0.020853	RTN4, RTN4R, NGFR	109	28	13528	13.29751	1	0.241878851	29.537
GOTERM_BP_FAT	GO:0042035~regulation of cytokine biosynthetic process	4	3.33333	0.021299	TICAM1, IRF1, BCL3, IGF2BP2	109	74	13528	6.7086536	1	0.244552438	30.069
GOTERM_BP_FAT	GO:0006915~apoptosis	11	9.16667	0.021975	RTN4, ARHGEF2, TIAM1, CDK11A, TICAM1, BUB1B, ITGB2, NGFR, ECT2, CD27, ARHGEF11	109	602	13528	2.2677924	1	0.249446724	30.867
GOTERM_CC_FAT	GO:0005694~chromosome	9	7.5	0.029065	ATRX, SUZ12, PPP2R5A, ZWINT, BUB1B, CTCF, MCM2, BUB3, RPA3	102	460	12782	2.4517903	0.99876	0.227034869	31.433
GOTERM_BP_FAT	GO:0009891~positive regulation of biosynthetic process	12	10	0.02279	CCNE1, EPAS1, TICAM1, GRIN1, EDN1, IRF1, BCL3, CTCF, CALR, CCNA2, ARHGEF11	109	695	13528	2.1429081	1	0.255593706	31.818

GOTERM_BP_FAT	GO:0002520~immune system development	7	5.83333	0.023191	EPAS1, MMP9, IRF1, BCL3, NFKB2, CD79A, BLNK	109	276	13528	3.1477197	1	0.257604647	32.281
GOTERM_BP_FAT	GO:0008016~regulation of heart contraction	4	3.33333	0.023628	PRKCA, EPAS1, DRD2, EDN1	109	77	13528	6.4472775	1	0.259928237	32.783
GOTERM_BP_FAT	GO:0012501~programmed cell death	11	9.16667	0.024056	RTN4, ARHGEF2, TIAM1, CDK11A, TICAM1, BUB1B, ITGB2, NGFR, ECT2, CD27, ARHGEF11	109	611	13528	2.2343879	1	0.262113594	33.27
GOTERM_CC_FAT	GO:0033256~I-kappaB/NF-kappaB complex	2	1.66667	0.031238	BCL3, NFKB2	102	4	12782	62.656863	0.99926	0.234188604	33.37
GOTERM_MF_FAT	GO:0003682~chromatin binding	5	4.16667	0.029512	ATRX, SUZ12, EZH2, ACTL6A, CTCF	102	150	12983	4.2428105	0.99997	0.614470251	33.615
GOTERM_BP_FAT	GO:0032880~regulation of protein localization	5	4.16667	0.024595	DRD2, PRKCI, BCL3, CDH2, CD27	109	138	13528	4.4967425	1	0.265307195	33.88
GOTERM_BP_FAT	GO:0006952~defense response	11	9.16667	0.025025	A2M, TFRC, AOX1, TICAM1, CXCL9, CCL19, BCL3, HLA-C, ITGB2, TAPBP, BLNK	109	615	13528	2.2198553	1	0.267420755	34.362

GOTERM_BP_FAT	GO:0007632~visual behavior	3	2.5	0.025259	SLC1A2, DRD2, GRIN1	109	31	13528	12.010654	1	0.267709448	34.624
GOTERM_MF_FAT	GO:0005088~Ras guanyl-nucleotide exchange factor activity	4	3.33333	0.031161	ARHGEF2, TIAM1, ECT2, ARHGEF11	102	88	12983	5.7856506	0.99998	0.602799917	35.141
GOTERM_BP_FAT	GO:0009611~response to wounding	10	8.33333	0.025807	A2M, SLC1A2, TFRC, AOX1, TICAM1, CXCL9, CCL19, ITGB2, NGFR, BLNK	109	530	13528	2.3416998	1	0.270835551	35.231
GOTERM_BP_FAT	GO:0048667~cell morphogenesis involved in neuron differentiation	6	5	0.026002	PRKCA, PARD3, NDN, DRD2, RTN4R, NGFR	109	209	13528	3.5629691	1	0.270745098	35.447
GOTERM_BP_FAT	GO:0051153~regulation of striated muscle cell differentiation	3	2.5	0.026805	EZH2, EDN1, MBNL3	109	32	13528	11.635321	1	0.276045612	36.325
GOTERM_MF_FAT	GO:0015078~hydrogen ion transmembrane transporter activity	4	3.33333	0.032994	UQCRC1, UQCRH, COX7C, UQCRQ	102	90	12983	5.6570806	0.99999	0.594763458	36.8
GOTERM_BP_FAT	GO:0048812~neuron projection morphogenesis	6	5	0.027916	PRKCA, PARD3, NDN, DRD2, RTN4R, NGFR	109	213	13528	3.4960589	1	0.28391833	37.522
GOTERM_BP_FAT	GO:0030030~cell projection organization	8	6.66667	0.027939	PRKCA, PARD3, NDN, DRD2, ITGA8, RTN4R, NGFR, GRIN3A	109	368	13528	2.6980455	1	0.282248491	37.547

GOTERM_CC_FAT	GO:0043197~dendritic spine	3	2.5	0.036142	SLC1A2, ITGA8, GRIN1	102	38	12782	9.8931889	0.99977	0.258021865	37.559
GOTERM_BP_FAT	GO:0046324~regulation of glucose import	3	2.5	0.028388	PRKCA, SLC1A2, PRKCI	109	33	13528	11.282736	1	0.284246022	38.024
GOTERM_MF_FAT	GO:0017048~Rho GTPase binding	3	2.5	0.035131	PARD6A, ARHGEF2, ECT2	102	38	12983	10.048762	1	0.591013892	38.684
GOTERM_MF_FAT	GO:0035258~steroid hormone receptor binding	3	2.5	0.035131	CCNE1, CALR, PRPF6	102	38	12983	10.048762	1	0.591013892	38.684
GOTERM_BP_FAT	GO:0031398~positive regulation of protein ubiquitination	4	3.33333	0.029578	ANAPC1, PSMB5, PSMA4, PSMB8	109	84	13528	5.9100044	1	0.292468797	39.273
GOTERM_CC_FAT	GO:0005768~endosome	7	5.83333	0.03859	LRP1, TFRC, PRKCI, HFE, CD79A, NGFR, B2M	102	315	12782	2.7847495	0.99987	0.265123903	39.558
GOTERM_BP_FAT	GO:0017148~negative regulation of translation	3	2.5	0.030007	PRKCA, IGF2BP2, CALR	109	34	13528	10.95089	1	0.294177182	39.718
GOTERM_BP_FAT	GO:0010827~regulation of glucose transport	3	2.5	0.030007	PRKCA, SLC1A2, PRKCI	109	34	13528	10.95089	1	0.294177182	39.718
GOTERM_MF_FAT	GO:0042054~histone methyltransferase activity	3	2.5	0.036846	SUZ12, EZH2, SETD2	102	39	12983	9.7911011	1	0.583543523	40.157
GOTERM_MF_FAT	GO:0019903~protein phosphatase binding	3	2.5	0.036846	LILRB1, GRIN3A, CDH2	102	39	12983	9.7911011	1	0.583543523	40.157
GOTERM_CC_FAT	GO:0030425~dendrite	5	4.16667	0.040166	SLC1A2, LRP1, DRD2, ITGA8, GRIN1	102	163	12782	3.8439793	0.99991	0.266694888	40.813

GOTERM_BP_FAT	GO:0002467~germinal center formation	2	1.66667	0.031557	BCL3, NFKB2	109	4	13528	62.055046	1	0.30503565	41.298
GOTERM_BP_FAT	GO:0045216~cell-cell junction organization	3	2.5	0.031663	PARD6A, PRKCI, CTNNA1	109	35	13528	10.638008	1	0.303991135	41.405
GOTERM_BP_FAT	GO:0009060~aerobic respiration	3	2.5	0.031663	SDHB, UQCRC1, UQCRH	109	35	13528	10.638008	1	0.303991135	41.405
GOTERM_BP_FAT	GO:0007205~activation of protein kinase C activity by G-protein coupled receptor protein signaling pathway	3	2.5	0.033354	PARD3, LRP1, EDN1	109	36	13528	10.342508	1	0.315609523	43.081
GOTERM_BP_FAT	GO:0007346~regulation of mitotic cell cycle	5	4.16667	0.033432	ZWINT, EDN1, BUB1B, CCNA2, BUB3	109	152	13528	4.0825688	1	0.314297651	43.158
GOTERM_BP_FAT	GO:0043434~response to peptide hormone stimulus	5	4.16667	0.034827	PRKCA, PRKCI, TFF1, PARP1, CCNA2	109	154	13528	4.0295484	1	0.323246879	44.505

GOTERM_CC_FAT	GO:0043228~non-membrane-bounded organelle	29	24.1667	0.04509	[illegible] PARD3, NDN, PPP2R5A, CTCF, IGF2BP2, GRIN3A, LIN28A, RPA3, ZNF207, CCNE1, MRPL15, SAFB, MBNL3, BUB3, ARHGDIB, ARHGEF2, GRIN1, MCM2, SLC9A3R1, CTNNA1, ARHGEF11, SUZ12, ATRX, ATF3, ZWINT, [illegible]	102	2596	12782	1.3998837	0.99997	0.286695882	44.582

GOTERM_CC_FAT	GO:0043232~intracellular non-membrane-bounded organelle	29	24.1667	0.04509	POLR2H, PARD3, NDN, PPP2R5A, CTCF, IGF2BP2, GRIN3A, LIN28A, RPA3, ZNF207, CCNE1, MRPL15, SAFB, MBNL3, BUB3, ARHGDIB, ARHGEF2, GRIN1, MCM2, SLC9A3R1, CTNNA1, ARHGEF11, SUZ12, ATRX, ATF3, ZWINT, ITGA8	102	2596	12782	1.3998837	0.99997	0.286695882	44.582
GOTERM_BP_FAT	GO:0030595~leukocyte chemotaxis	3	2.5	0.035079	PRKCA, ITGB2, ITGAM	109	37	13528	10.06298	1	0.323253717	44.746
GOTERM_CC_FAT	GO:0043275~respiratory chain complex III	2	1.66667	0.046493	UQCRC1, UQCRH	102	6	12782	41.771242	0.99998	0.286604835	45.615
GOTERM_CC_FAT	GO:0005750~mitochondrial respiratory chain complex III	2	1.66667	0.046493	UQCRC1, UQCRH	102	6	12782	41.771242	0.99998	0.286604835	45.615

GOTERM_BP_FAT	GO:0010557~positive regulation of macromolecule biosynthetic process	11	9.16667	0.036004	CCNE1, EPAS1, TICAM1, GRIN1, IRF1, BCL3, CTCF, CALR, CCNA2, ARHGEF11, PRPF6	109	654	13528	2.0874786	1	0.32836506	45.619
GOTERM_BP_FAT	GO:0000075~cell cycle checkpoint	4	3.33333	0.036246	ZWINT, BUB1B, CCNA2, BUB3	109	91	13528	5.4553886	1	0.328252658	45.845
GOTERM_MF_FAT	GO:0019902~phosphatase binding	3	2.5	0.045883	LILRB1, GRIN3A, CDH2	102	44	12983	8.6784759	1	0.642078516	47.396
GOTERM_BP_FAT	GO:0051147~regulation of muscle cell differentiation	3	2.5	0.038632	EZH2, EDN1, MBNL3	109	39	13528	9.5469301	1	0.3439762	48.03
GOTERM_BP_FAT	GO:0006479~protein amino acid methylation	3	2.5	0.038632	SUZ12, EZH2, SETD2	109	39	13528	9.5469301	1	0.3439762	48.03
GOTERM_BP_FAT	GO:0008213~protein amino acid alkylation	3	2.5	0.038632	SUZ12, EZH2, SETD2	109	39	13528	9.5469301	1	0.3439762	48.03
GOTERM_BP_FAT	GO:0060326~cell chemotaxis	3	2.5	0.038632	PRKCA, ITGB2, ITGAM	109	39	13528	9.5469301	1	0.3439762	48.03
GOTERM_BP_FAT	GO:0014075~response to amine stimulus	3	2.5	0.038632	SLC1A2, DRD2, GRIN1	109	39	13528	9.5469301	1	0.3439762	48.03
GOTERM_BP_FAT	GO:0032270~positive regulation of cellular protein metabolic process	6	5	0.03882	PRKCA, ANAPC1, PSMB5, PSMA4, BCL3, PSMB8	109	233	13528	3.195968	1	0.343375887	48.198
GOTERM_MF_FAT	GO:0015077~monovalent inorganic cation transmembrane transporter activity	4	3.33333	0.047357	UQCRC1, UQCRH, COX7C, UQCRQ	102	104	12983	4.8955505	1	0.631691981	48.497

GOTERM_BP_FAT	GO:0019932~second-messenger-mediated signaling	6	5	0.040035	TYMS, DRD2, ZWINT, EDN1, MTNR1B, BUB1B	109	235	13528	3.1687683	1	0.350158478	49.276
GOTERM_BP_FAT	GO:0030097~hemopoiesis	6	5	0.040651	EPAS1, MMP9, IRF1, BCL3, CD79A, BLNK	109	236	13528	3.1553413	1	0.352566525	49.814
GOTERM_MF_FAT	GO:0016763~transferase activity, transferring pentosyl groups	3	2.5	0.049703	TYMP, MTAP, PARP1	102	46	12983	8.3011509	1	0.628904488	50.205
GOTERM_CC_FAT	GO:0045177~apical part of cell	5	4.16667	0.053362	PARD3, ITGA8, PRKCI, HFE, SLC9A3R1	102	179	12782	3.5003834	1	0.314236778	50.42
GOTERM_MF_FAT	GO:0001846~opsonin binding	2	1.66667	0.053213	CALR, ITGAM	102	7	12983	36.366947	1	0.634792498	52.662
GOTERM_MF_FAT	GO:0016725~oxidoreductase activity, acting on CH or CH2 groups	2	1.66667	0.053213	RRM2, AOX1	102	7	12983	36.366947	1	0.634792498	52.662
GOTERM_BP_FAT	GO:0006511~ubiquitin-dependent protein catabolic process	6	5	0.04447	ANAPC1, PSMB5, PSMA4, BUB1B, BUB3, PSMB8	109	242	13528	3.0771097	1	0.377001674	53.032
GOTERM_BP_FAT	GO:0051247~positive regulation of protein metabolic process	6	5	0.045127	PRKCA, ANAPC1, PSMB5, PSMA4, BCL3, PSMB8	109	243	13528	3.0644467	1	0.379389125	53.565

GOTERM_BP_FAT	GO:0000904~cell morphogenesis involved in differentiation	6	5	0.045789	PRKCA, PARD3, NDN, DRD2, RTN4R, NGFR	109	244	13528	3.0518875	1	0.381776564	54.097
GOTERM_BP_FAT	GO:0048858~cell projection morphogenesis	6	5	0.046458	PRKCA, PARD3, NDN, DRD2, RTN4R, NGFR	109	245	13528	3.0394308	1	0.384163851	54.629
GOTERM_BP_FAT	GO:0006122~mitochondrial electron transport, ubiquinol to cytochrome c	2	1.66667	0.046963	UQCRC1, UQCRH	109	6	13528	41.370031	1	0.385452025	55.027
GOTERM_CC_FAT	GO:0032589~neuron projection membrane	2	1.66667	0.061509	SLC1A2, ITGA8	102	8	12782	31.328431	1	0.345470435	55.61
GOTERM_CC_FAT	GO:0017146~N-methyl-D-aspartate selective glutamate receptor complex	2	1.66667	0.061509	GRIN1, GRIN3A	102	8	12782	31.328431	1	0.345470435	55.61
GOTERM_MF_FAT	GO:0008276~protein methyltransferase activity	3	2.5	0.057672	SUZ12, EZH2, SETD2	102	50	12983	7.6370588	1	0.646381958	55.622
GOTERM_BP_FAT	GO:0022403~cell cycle phase	8	6.66667	0.047834	ANAPC1, CCNE1, ARHGEF2, CDK11A, ZWINT, BUB1B, CCNA2, BUB3	109	414	13528	2.3982626	1	0.389080006	55.704

GOTERM_BP_FAT	GO:0000279~M phase	7	5.83333	0.048112	ANAPC1, ARHGEF2, CDK11A, ZWINT, BUB1B, CCNA2, BUB3	109	329	13528	2.6406402	1	0.388856114	55.919
GOTERM_BP_FAT	GO:0070271~protein complex biogenesis	9	7.5	0.048911	POLR2H, PARD3, UQCRH, RRM2, HFE, CDH2, SLC9A3R1, CALR, TAPBP	109	505	13528	2.211863	1	0.391948423	56.53
GOTERM_BP_FAT	GO:0006461~protein complex assembly	9	7.5	0.048911	POLR2H, PARD3, UQCRH, RRM2, HFE, CDH2, SLC9A3R1, CALR, TAPBP	109	505	13528	2.211863	1	0.391948423	56.53
GOTERM_BP_FAT	GO:0010627~regulation of protein kinase cascade	6	5	0.049189	AMBP, DRD2, TICAM1, EDN1, ECT2, CD27	109	249	13528	2.9906046	1	0.391709555	56.74
GOTERM_BP_FAT	GO:0030098~lymphocyte differentiation	4	3.33333	0.04931	IRF1, BCL3, CD79A, BLNK	109	103	13528	4.8198094	1	0.390487779	56.831
GOTERM_BP_FAT	GO:0006259~DNA metabolic process	9	7.5	0.049379	ATRX, TYMS, TYMP, RRM2, CTCF, MCM2, PARP1, MCM6, RPA3	109	506	13528	2.2074918	1	0.388954877	56.883
GOTERM_BP_FAT	GO:0050768~negative regulation of neurogenesis	3	2.5	0.050055	RTN4, RTN4R, NGFR	109	45	13528	8.2740061	1	0.391211812	57.39

GOTERM_BP_FAT	GO:0030198~extracellular matrix organization	4	3.33333	0.05049	MMP9, ITGA8, BCL3, NFKB2	109	104	13528	4.7734651	1	0.391950238	57.713
GOTERM_BP_FAT	GO:0043627~response to estrogen stimulus	4	3.33333	0.051683	PRKCA, CCNE1, TFF1, CCNA2	109	105	13528	4.7280035	1	0.397296549	58.587
GOTERM_MF_FAT	GO:0003712~transcription cofactor activity	7	5.83333	0.063724	CCNE1, ATF3, EPAS1, WDR77, CTCF, NFKB2, PRPF6	102	363	12983	2.4545184	1	0.66627044	59.366
GOTERM_BP_FAT	GO:0032990~cell part morphogenesis	6	5	0.054195	PRKCA, PARD3, NDN, DRD2, RTN4R, NGFR	109	256	13528	2.9088303	1	0.410376303	60.372
GOTERM_MF_FAT	GO:0005529~sugar binding	5	4.16667	0.065616	TALDO1, CALR, KLRD1, CANX, KLRC1	102	195	12983	3.2637004	1	0.660310104	60.474
GOTERM_BP_FAT	GO:0030147~natriuresis	2	1.66667	0.054575	DRD2, EDN1	109	7	13528	35.460026	1	0.410650637	60.636
GOTERM_BP_FAT	GO:0048387~negative regulation of retinoic acid receptor signaling pathway	2	1.66667	0.054575	EZH2, CALR	109	7	13528	35.460026	1	0.410650637	60.636
GOTERM_MF_FAT	GO:0032403~protein complex binding	5	4.16667	0.0666	AMBP, CCNE1, UQCRC1, UQCRH, CALR	102	196	12983	3.2470488	1	0.649645364	61.04
GOTERM_BP_FAT	GO:0040008~regulation of growth	7	5.83333	0.055428	RTN4, NDN, CDK11A, DRD2, ACTL6A, PARP1, ARHGEF11	109	341	13528	2.5477145	1	0.413673212	61.222

GOTERM_CC_FAT	GO:0044454~nuclear chromosome part	4	3.33333	0.072186	ATRX, SUZ12, MCM2, RPA3	102	122	12782	4.1086467	1	0.384877956	61.655
GOTERM_CC_FAT	GO:0044451~nucleoplasm part	9	7.5	0.072187	POLR2H, SUZ12, PPP1CA, EPAS1, PPP1R8, EZH2, CCNL1	102	555	12782	2.0321145	1	0.376525595	61.656
GOTERM_BP_FAT	GO:0010721~negative regulation of cell development	3	2.5	0.056167	RTN4, RTN4R, NGFR	109	48	13528	7.7568807	1	0.415997629	61.723
GOTERM_BP_FAT	GO:0030183~B cell differentiation	3	2.5	0.056167	BCL3, CD79A, BLNK	109	48	13528	7.7568807	1	0.415997629	61.723
GOTERM_BP_FAT	GO:0042108~positive regulation of cytokine biosynthetic	3	2.5	0.056167	TICAM1, IRF1, BCL3	109	48	13528	7.7568807	1	0.415997629	61.723
GOTERM_MF_FAT	GO:0019958~C-X-C chemokine binding	2	1.66667	0.067895	A2M, CXCR3	102	9	12983	28.285403	1	0.641331324	61.773
GOTERM_BP_FAT	GO:0009615~response to virus	4	3.33333	0.056593	LILRB1, TICAM1, CCL19, BCL3	109	109	13528	4.5544988	1	0.416493949	62.009
GOTERM_CC_FAT	GO:0042611~MHC protein complex	3	2.5	0.074566	HFE, HLA-C, B2M	102	57	12782	6.5954592	1	0.378379993	62.894
GOTERM_BP_FAT	GO:0007163~establishment or maintenance of cell polarity	3	2.5	0.05826	PARD3, PRKCI, ARHGEF11	109	49	13528	7.598577	1	0.424002593	63.109
GOTERM_MF_FAT	GO:0003823~antigen binding	3	2.5	0.070384	PPP2R1B, KIR2DL3, TAPBP	102	56	12983	6.8188025	1	0.640042867	63.145
GOTERM_BP_FAT	GO:0007611~learning or memory	4	3.33333	0.059128	PRKCA, DRD2, ITGA8, GRIN1	109	111	13528	4.4724357	1	0.426909538	63.67

GOTERM_CC_FAT	GO:0031519~PcG protein complex	2	1.66667	0.076292	SUZ12, EZH2	102	10	12782	25.062745	1	0.377534567	63.77
GOTERM_BP_FAT	GO:0032102~negative regulation of response to external stimulus	3	2.5	0.06038	DRD2, GRIN1, GRIN3A	109	50	13528	7.4466055	1	0.431880331	64.464
GOTERM_BP_FAT	GO:0030182~neuron differentiation	8	6.66667	0.061111	PRKCA, PARD3, NDN, DRD2, RTN4R, PRKCI, NGFR, GRIN3A	109	438	13528	2.266851	1	0.433932047	64.921
GOTERM_BP_FAT	GO:0008219~cell death	11	9.16667	0.061124	RTN4, ARHGEF2, TIAM1, CDK11A, TICAM1, BUB1B, ITGB2, NGFR, ECT2, CD27, ARHGEF11	109	719	13528	1.8987636	1	0.432070924	64.929
GOTERM_BP_FAT	GO:0045935~positive regulation of nucleobase, nucleoside, nucleotide and nucleic acid metabolic process	10	8.33333	0.061555	CCNE1, EPAS1, GRIN1, IRF1, BCL3, CTCF, CALR, CCNA2, ARHGEF11, PRPF6	109	624	13528	1.9889438	1	0.432483785	65.195
GOTERM_BP_FAT	GO:0009263~deoxyribonucleotide biosynthetic process	2	1.66667	0.062127	TYMS, RRM2	109	8	13528	31.027523	1	0.433645658	65.546
GOTERM_BP_FAT	GO:0030146~diuresis	2	1.66667	0.062127	DRD2, EDN1	109	8	13528	31.027523	1	0.433645658	65.546

GOTERM_BP_FAT	GO:0031401~positive regulation of protein modification process	5	4.16667	0.062692	PRKCA, ANAPC1, PSMB5, PSMA4, PSMB8	109	187	13528	3.3184517	1	0.43476097	65.89
GOTERM_BP_FAT	GO:0022900~electron transport chain	4	3.33333	0.06303	SDHB, UQCRC1, UQCRH, UQCRQ	109	114	13528	4.3547401	1	0.434660647	66.093
GOTERM_BP_FAT	GO:0016265~death	11	9.16667	0.063438	RTN4, ARHGEF2, TIAM1, CDK11A, TICAM1, BUB1B, ITGB2, NGFR, ECT2, CD27, ARHGEF11	109	724	13528	1.8856506	1	0.434926432	66.337
GOTERM_BP_FAT	GO:0044265~cellular macromolecule catabolic process	11	9.16667	0.063907	ANAPC1, PSMB5, FBXW7, PPP1R8, PSMA4, BUB1B, NGFR, LIN28A, BUB3	109	725	13528	1.8830497	1	0.435514995	66.617
GOTERM_CC_FAT	GO:0000502~proteasome complex	3	2.5	0.083806	PSMB5, PSMA4, PSMB8	102	61	12782	6.1629701	1	0.399176801	67.365
GOTERM_BP_FAT	GO:0040012~regulation of locomotion	5	4.16667	0.067703	PRKCA, RTN4, DRD2, MMP9, EDN1	109	192	13528	3.2320336	1	0.453128019	68.796
GOTERM_BP_FAT	GO:0048545~response to steroid hormone stimulus	5	4.16667	0.067703	PRKCA, CCNE1, A2M, TFF1, CCNA2	109	192	13528	3.2320336	1	0.453128019	68.796

GOTERM_BP_FAT	GO:0051270~regulation of cell motion	5	4.16667	0.06873	RTN4, PARD3, DRD2, MMP9, EDN1	109	193	13528	3.2152874	1	0.456389582	69.362
GOTERM_BP_FAT	GO:0043279~response to alkaloid	3	2.5	0.069116	UQCRC1, DRD2, GRIN1	109	54	13528	6.8950051	1	0.456424184	69.572
GOTERM_BP_FAT	GO:0035235~ionotropic glutamate receptor signaling pathway	2	1.66667	0.069619	GRIN1, GRIN3A	109	9	13528	27.58002	1	0.45703823	69.844
GOTERM_BP_FAT	GO:0048385~regulation of retinoic acid receptor signaling pathway	2	1.66667	0.069619	EZH2, CALR	109	9	13528	27.58002	1	0.45703823	69.844
GOTERM_BP_FAT	GO:0016337~cell-cell adhesion	6	5	0.070075	PARD3, ITGA8, CD2, ITGB2, CDH2, ITGAM	109	276	13528	2.6980455	1	0.457417562	70.089
GOTERM_BP_FAT	GO:0016477~cell migration	6	5	0.070075	PRKCA, NDN, DRD2, ITGB2, CDH2, ITGAM	109	276	13528	2.6980455	1	0.457417562	70.089
GOTERM_MF_FAT	GO:0003713~transcription coactivator activity	5	4.16667	0.085608	CCNE1, EPAS1, WDR77, NFKB2, PPRF6	102	214	12983	2.9739326	1	0.700235738	70.596
GOTERM_BP_FAT	GO:0051094~positive regulation of developmental process	6	5	0.071791	PRKCA, CCNE1, TIAM1, DRD2, NGFR, CD27	109	278	13528	2.6786351	1	0.463912205	70.993
GOTERM_BP_FAT	GO:0014070~response to organic cyclic substance	4	3.33333	0.072586	PRKCA, CCNE1, DRD2, GRIN1	109	121	13528	4.102813	1	0.46587375	71.403
GOTERM_BP_FAT	GO:0070201~regulation of establishment of protein localization	4	3.33333	0.072586	DRD2, PRKCI, BCL3, CD27	109	121	13528	4.102813	1	0.46587375	71.403

GOTERM_BP_FAT	GO:0009725~response to hormone stimulus	7	5.83333	0.073483	PRKCA, CCNE1, A2M, PRKCI, TFF1, PARP1, CCNA2	109	367	13528	2.3672225	1	0.468295566	71.859
GOTERM_BP_FAT	GO:0016570~histone modification	4	3.33333	0.074001	SUZ12, EZH2, ACTL6A, SETD2	109	122	13528	4.0691833	1	0.468898951	72.119
GOTERM_MF_FAT	GO:0016594~glycine binding	2	1.66667	0.089497	GRIN1, GRIN3A	102	12	12983	21.214052	1	0.703402138	72.26
GOTERM_BP_FAT	GO:0007243~protein kinase cascade	7	5.83333	0.075761	PRKCA, DRD2, TICAM1, EDN1, BCL3, CD27, TANK	109	370	13528	2.3480288	1	0.475292879	72.986
GOTERM_BP_FAT	GO:0050900~leukocyte migration	3	2.5	0.075923	PRKCA, ITGB2, ITGAM	109	57	13528	6.5321101	1	0.474199089	73.065
GOTERM_BP_FAT	GO:0034330~cell junction organization	3	2.5	0.075923	PARD6A, PRKCI, CTNNA1	109	57	13528	6.5321101	1	0.474199089	73.065
GOTERM_CC_FAT	GO:0045120~pronucleus	2	1.66667	0.098035	EZH2, CCNA2	102	13	12782	19.279035	1	0.443196009	73.287
GOTERM_MF_FAT	GO:0005506~iron ion binding	6	5	0.092166	AMBP, SDHB, PPP1CA, RRM2, AOX1, HFE	102	308	12983	2.4795646	1	0.701404669	73.352
GOTERM_BP_FAT	GO:0014821~phasic smooth muscle contraction	2	1.66667	0.077051	DRD2, EDN1	109	10	13528	24.822018	1	0.477564693	73.606
GOTERM_BP_FAT	GO:0003091~renal water homeostasis	2	1.66667	0.077051	DRD2, EDN1	109	10	13528	24.822018	1	0.477564693	73.606
GOTERM_BP_FAT	GO:0016569~covalent chromatin modification	4	3.33333	0.079785	SUZ12, EZH2, ACTL6A, SETD2	109	126	13528	3.9400029	1	0.488116161	74.875

GOTERM_BP_FAT	GO:0042110~T cell activation	4	3.33333	0.079785	CD2, IRF1, BCL3, ITGAM	109	126	13528	3.9400029	1	0.488116161	74.875
GOTERM_BP_FAT	GO:0050769~positive regulation of neurogenesis	3	2.5	0.080574	TIAM1, DRD2, NGFR	109	59	13528	6.3106826	1	0.489796765	75.231
GOTERM_MF_FAT	GO:0019992~diacylglycerol binding	3	2.5	0.098152	PRKCA, ARHGEF2, PRKCI	102	68	12983	5.6154844	1	0.712587617	75.657
GOTERM_BP_FAT	GO:0045941~positive regulation of transcription	9	7.5	0.081589	CCNE1, EPAS1, GRIN1, IRF1, BCL3, CTCF, CCNA2, ARHGEF11, PRPF6	109	564	13528	1.9804802	1	0.492454081	75.681
GOTERM_BP_FAT	GO:0006325~chromatin organization	7	5.83333	0.082032	SUZ12, SAFB, EZH2, ACTL6A, CTCF, MCM2, SETD2	109	378	13528	2.298335	1	0.492571831	75.875

GOTERM_BP_FAT	GO:0045449~regulation of transcription	28	23.3333	0.082913	NDN, EZH2, CTCF, NFKB2, CALR, LIN28A, ZNF207, CCNE1, SAFB, TICAM1, PHTF1, BCL3, ACTL6A, CCNA2, EPAS1, GRIN1, CCNL1, MCM2, PRPF6, ARHGEF11, MCM6, SUZ12, ATRX, ATF3, CDK11A,	109	2601	13528	1.3360564	1	0.49459743	76.257
GOTERM_BP_FAT	GO:0050891~multicellular organismal water homeostasis	2	1.66667	0.084425	DRD2, EDN1	109	11	13528	22.565471	1	0.499311063	76.899
GOTERM_BP_FAT	GO:0002478~antigen processing and presentation of exogenous peptide antigen	2	1.66667	0.084425	TAPBP, B2M	109	11	13528	22.565471	1	0.499311063	76.899
GOTERM_BP_FAT	GO:0045893~positive regulation of transcription, DNA-dependent	8	6.66667	0.087115	CCNE1, EPAS1, GRIN1, IRF1, BCL3, CTCF, ARHGEF11, PRPF6	109	477	13528	2.081511	1	0.508909804	78.001

GOTERM_BP_FAT	GO:0046578~regulation of Ras protein signal transduction	5	4.16667	0.087397	ARHGEF2, TIAM1, CDH2, ECT2, ARHGEF11	109	210	13528	2.9550022	1	0.508277259	78.114
GOTERM_BP_FAT	GO:0050905~neuromuscular process	3	2.5	0.087712	DRD2, GRIN1, GRIN3A	109	62	13528	6.005327	1	0.507788641	78.239
GOTERM_BP_FAT	GO:0048514~blood vessel morphogenesis	5	4.16667	0.088565	RTN4, TYMP, EPAS1, EDN1, CDH2	109	211	13528	2.9409974	1	0.509533795	78.574
GOTERM_BP_FAT	GO:0010608~posttranscriptional regulation of gene expression	5	4.16667	0.088565	PRKCA, BCL3, IGF2BP2, LIN28A, CALR	109	211	13528	2.9409974	1	0.509533795	78.574
GOTERM_BP_FAT	GO:0051254~positive regulation of RNA metabolic process	8	6.66667	0.090094	CCNE1, EPAS1, GRIN1, IRF1, BCL3, CTCF, ARHGEF11, PRPF6	109	481	13528	2.0642011	1	0.514021848	79.164
GOTERM_BP_FAT	GO:0010039~response to iron ion	2	1.66667	0.09174	TFRC, TFF1	109	12	13528	20.685015	1	0.518910093	79.781
GOTERM_BP_FAT	GO:0051482~elevation of cytosolic calcium ion concentration during G-protein signaling, coupled to IP3 second messenger (phospholipase C	2	1.66667	0.09174	DRD2, EDN1	109	12	13528	20.685015	1	0.518910093	79.781
GOTERM_BP_FAT	GO:0051235~maintenance of location	3	2.5	0.092573	PDIA3, BCL3, CALR	109	64	13528	5.8176606	1	0.520466388	80.087

GOTERM_BP_FAT	GO:0022904~respiratory electron transport chain	3	2.5	0.092573	SDHB, UQCRC1, UQCRH	109	64	13528	5.8176606	1	0.520466388	80.087
GOTERM_BP_FAT	GO:0031668~cellular response to extracellular stimulus	3	2.5	0.092573	CCNE1, SLC1A2, HFE	109	64	13528	5.8176606	1	0.520466388	80.087
GOTERM_BP_FAT	GO:0010628~positive regulation of gene expression	9	7.5	0.092966	CCNE1, EPAS1, GRIN1, IRF1, BCL3, CTCF, CCNA2, ARHGEF11, PRPF6	109	581	13528	1.9225315	1	0.520259134	80.23
GOTERM_BP_FAT	GO:0009057~macromolecule catabolic process	11	9.16667	0.09386	ANAPC1, PSMB5, FBXW7, PPP1R8, PSMA4, BUB1B, NGFR, LIN28A, BUB3	109	781	13528	1.7480295	1	0.522036758	80.551
GOTERM_BP_FAT	GO:0046328~regulation of JNK cascade	3	2.5	0.095032	AMBP, EDN1, CD27	109	65	13528	5.7281581	1	0.524884287	80.965
GOTERM_BP_FAT	GO:0021537~telencephalon development	3	2.5	0.095032	SLC1A2, DRD2, GRIN1	109	65	13528	5.7281581	1	0.524884287	80.965
GOTERM_BP_FAT	GO:0044093~positive regulation of molecular function	9	7.5	0.096479	ANAPC1, PSMB5, PARD3, LRP1, DRD2, PSMA4, TICAM1, EDN1, PSMB8	109	586	13528	1.9061277	1	0.528756626	81.464
GOTERM_BP_FAT	GO:0006417~regulation of translation	4	3.33333	0.09666	PRKCA, BCL3, IGF2BP2, CALR	109	137	13528	3.6236523	1	0.527705297	81.526

GOTERM_BP_FAT	GO:0042127~regulation of cell proliferation	11	9.16667	0.0975	PRKCA, SUZ12, ARHGEF2, ATF3, NDN, DRD2, TICAM1, EDN1, NGFR	109	787	13528	1.7347027	1	0.529191303	81.809
GOTERM_BP_FAT	GO:0009416~response to light stimulus	4	3.33333	0.098261	SLC1A2, DRD2, GRIN1, BCL3	109	138	13528	3.597394	1	0.530364957	82.062
GOTERM_BP_FAT	GO:0048870~cell motility	6	5	0.099233	PRKCA, NDN, DRD2, ITGB2, CDH2, ITGAM	109	307	13528	2.4256044	1	0.532319349	82.381
GOTERM_BP_FAT	GO:0051674~localization of cell	6	5	0.099233	PRKCA, NDN, DRD2, ITGB2, CDH2, ITGAM	109	307	13528	2.4256044	1	0.532319349	82.381
GOTERM_BP_FAT	GO:0009991~response to extracellular stimulus	5	4.16667	0.099416	CCNE1, A2M, SLC1A2, TFRC, HFE	109	220	13528	2.8206839	1	0.531289971	82.44
KEGG_PATHWAY	hsa04612:Antigen processing and presentation	10	8.33333	5.32E-06	PDIA3, HLA-C, KIR2DL3, CALR, KLRD1, CANX, KIR3DL1, KLRC1, TAPBP, B2M	82	83	5085	7.4713488	4.95E-04	4.95E-04	0.0058
KEGG_PATHWAY	hsa05332:Graft-versus-host disease	5	4.16667	0.003218	HLA-C, KIR2DL3, KLRD1, KIR3DL1, KLRC1	82	39	5085	7.9502814	0.25898	0.139174173	3.4667

KEGG_PATHWAY	hsa04110:Cell cycle	7	5.83333	0.01413	ANAPC1, CCNE1, BUB1B, MCM2, CCNA2, BUB3	82	125	5085	3.4726829	0.73379	0.356710851	14.426
KEGG_PATHWAY	hsa04514:Cell adhesion molecules (CAMs)	7	5.83333	0.018071	ITGA8, CD2, HLA-C, ITGB2, CDH2, JAM3, ITGAM	82	132	5085	3.2885255	0.81657	0.345566557	18.097
KEGG_PATHWAY	hsa04650:Natural killer cell mediated cytotoxicity	7	5.83333	0.01869	PRKCA, HLA-C, ITGB2, KIR2DL3, KLRD1, KIR3DL1, KLRC1	82	133	5085	3.2637997	0.82702	0.2959559	18.661
KEGG_PATHWAY	hsa04530:Tight junction	7	5.83333	0.019323	PRKCA, PARD6A, PPP2R1B, PARD3, PRKCI, CTNNA1	82	134	5085	3.239443	0.8371	0.260983123	19.233
KEGG_PATHWAY	hsa04670:Leukocyte transendothelial migration	6	5	0.039153	PRKCA, MMP9, ITGB2, CTNNA1, JAM3, ITGAM	82	118	5085	3.1531625	0.97563	0.411765162	35.418
KEGG_PATHWAY	hsa05010:Alzheimer's disease	7	5.83333	0.044539	SDHB, LRP1, UQCRC1, UQCRH, GRIN1, COX7C	82	163	5085	2.6631004	0.98555	0.411186082	39.273

KEGG_PATHWAY	hsa05016:Huntington's disease	7	5.83333	0.066038	POLR2H, SDHB, UQCRC1, UQCRH, GRIN1	82	180	5085	2.4115854	0.99826	0.506369956	52.665
KEGG_PATHWAY	hsa04114:Oocyte meiosis	5	4.16667	0.097438	ANAPC1, PPP2R1B, CCNE1, PPP1CA, PPP2R5A	82	110	5085	2.8187361	0.99993	0.614576108	67.447
